# KIF7 Controls the Proliferation of Cells of the Respiratory Airway through Distinct Microtubule Dependent Mechanisms

**DOI:** 10.1371/journal.pgen.1005525

**Published:** 2015-10-06

**Authors:** Garry L. Coles, Laurel A. Baglia, Kate G. Ackerman

**Affiliations:** 1 Department of Biomedical Genetics, University of Rochester Medical Center, Rochester, New York, United States of America; 2 Department of Pediatrics, University of Rochester Medical Center, Rochester, New York, United States of America; 3 Center for Pediatric Biomedical Research, University of Rochester Medical Center, Rochester, New York, United States of America; Washington University School of Medicine, UNITED STATES

## Abstract

The cell cycle must be tightly coordinated for proper control of embryonic development and for the long-term maintenance of organs such as the lung. There is emerging evidence that *Kinesin family member 7* (*Kif7*) promotes *Hedgehog* (*Hh*) signaling during embryonic development, and its misregulation contributes to diseases such as ciliopathies and cancer. *Kif7* encodes a microtubule interacting protein that controls *Hh* signaling through regulation of microtubule dynamics within the primary cilium. However, whether *Kif7* has a function in nonciliated cells remains largely unknown. The role *Kif7* plays in basic cell biological processes like cell proliferation or cell cycle progression also remains to be elucidated. Here, we show that *Kif7* is required for coordination of the cell cycle, and inactivation of this gene leads to increased cell proliferation *in vivo* and *in vitro*. Immunostaining and transmission electron microscopy experiments show that *Kif7* ^*dda/dda*^ mutant lungs are hyperproliferative and exhibit reduced alveolar epithelial cell differentiation. KIF7 depleted C3H10T1/2 fibroblasts and *Kif7* ^*dda/dda*^ mutant mouse embryonic fibroblasts have increased growth rates at high cellular densities, suggesting that *Kif7* may function as a general regulator of cellular proliferation. We ascertained that in G1, *Kif7* and microtubule dynamics regulate the expression and activity of several components of the cell cycle machinery known to control entry into S phase. Our data suggest that *Kif7* may function to regulate the maintenance of the respiratory airway architecture by controlling cellular density, cell proliferation, and cycle exit through its role as a microtubule associated protein.

## Author Summary

Respiratory diseases such as lung cancer, COPD, and asthma are the second leading cause of death in the United States. These diseases are heterogeneous and arise from genetic factors, environmental hazards, or developmental abnormalities that persist throughout life. An increased understanding of the genes and cellular mechanisms regulating respiratory system homeostasis and regeneration should provide information for the development of future therapeutics. We show that the gene *Kif7* regulates cell proliferation, cellular density, and intracellular signaling within the epithelial and mesenchymal cells of the respiratory airway. We expand on the known role for *Kif7* in regulating microtubule architecture within ciliated cells by showing that this protein regulates cell signaling in non-ciliated secretory cells. Furthermore, we show that microtubules function to regulate the abundance and activity of several factors known to be required for proper cell cycle timing. We propose that *Kif7* and microtubule dynamics hone cellular signaling necessary for control of the balance between cell proliferation and cell cycle exit, and we provide evidence that *Kif7* has a critical role in the maintenance of the respiratory system in postnatal life.

## Introduction

The alveolar epithelium needs to be maintained throughout life as it can be damaged by pathogens, environmental toxins, and various disease processes [1,2]. When the alveolar epithelium is damaged, these cell populations need to be replenished to maintain proper lung function [3]. The distal respiratory airway contains alveoli lined with specialized epithelial cells (type 1 and type 2 alveolar epithelial cells (AECs)) as well as a heterogeneous population of mesenchymal cells required to supply blood for gas exchange and to support a variety of metabolic activities. Type 1 AECs are large squamous cells that express podoplanin (PDPN) and share a common basement membrane with capillaries, allowing for efficient gas exchange. Type 2 AECs are secretory cells marked by the expression of surfactant proteins such as surfactant associated protein c (SFTPC), a lipoprotein complex which reduces alveolar surface tension and prevents alveolar collapse. The mesenchymal cells include fibroblasts that produce elastin, a protein important for lung recoil upon exhalation [3]. Recent work suggests that type 2 AECs function as adult stem/progenitor cells to replace injured AEC 1 and AEC 2 cells [4]. An increased understanding of the cellular mechanisms regulating alveolar epithelial cell proliferation will be valuable for the development of therapeutic interventions for lung diseases.

The regulation of cell division is critical for both normal development and injury repair. During the cell cycle, the centrosome is duplicated and the genome is replicated prior to mitosis. These events are controlled by cyclins that activate cyclin dependent kinases (CDKs) [5]. Cell proliferation is influenced by several factors including growth factor availability and cellular density. Growth factors are necessary for the transition from the G1 into S phase of the cell cycle. When cells grown in culture are deprived of growth factors, they arrest in G1 at the restriction point. Passage through the restriction point requires growth factor mediated induction of cyclin d1 and activation of CDK4/6. All subsequent stages of the cell cycle are believed to be growth factor independent [6]. Cyclin d and CDK4/6 work with cyclin e/CDK2 to phosphorylate and inactivate retinoblastoma (RB) to allow passage into S phase [7]. CDK2 also serves to regulate centrosome duplication in S phase via candidate molecules such as nucleophosmin (NPM1) [8,9]. Following centrosome duplication and DNA synthesis, cyclin a/CDK1/2 and cyclin b/CDK1 function to coordinate cell division. Successful completion of cell division requires the degradation of cyclin b/CDK1 by the anaphase promoting complex (APC). Defects in cell cycle entry and cell cycle exit are hallmarks of diseases such as cancer [5–7].

*Hedgehog (Hh)* signaling is a potent regulator of the development and maintenance of numerous tissues [10]. Kinesin family member 7 (KIF7) is a microtubule interacting protein that functions to regulate *Hh* signaling by maintaining the architecture of the primary cilium [11], a complex microtubule based organelle with many signal transduction functions [12,13]. Deletion of *Kif7* results in a defect in microtubule processing within the primary cilium and misactivation of the *Gli* family transcription factors (GLI 1–3, the *Hh* effector molecules) [10]. We have determined that KIF7 is expressed in cells containing primary cilia and in mature AEC cells that lack primary cilia. KIF7s function in nonciliated cells remains underexplored.

We previously identified a loss of function allele of *Kif7* (*Kif7* ^*dda*^, abbreviated here as *Kif7* ^*dd*^, or *Kif7* mutant) in a forward genetic screen aimed at recovering novel genes and mouse mutations that affect respiratory and cardiac function at birth. Analysis of both this mouse mutant allele and the *Kif7* ^*maki/maki*^ mutant embryos revealed that *Kif7* mutant embryos have tissue patterning and tissue hyperplasia phenotypes [14]. Therefore, we set out to determine if *Kif7* has a cell autonomous role in the regulation of cellular proliferation. We considered if this gene functions to restrain cell proliferation solely via its role as a regulator of *Hh* signaling. Finely, we explored whether this molecule functions to regulate the maintenance of the epithelial and mesenchymal cells of the distal lung, as these cells are often damaged in diseases.

Using a combination of molecular, cell biological, and mouse genetic techniques, we determined that *Kif7* functions to regulate cellular proliferation and the growth potential of fibroblasts in the mouse. Our analysis of the *Kif7* ^*dd/dd*^ pulmonary phenotype and KIF7 depleted mouse lung fibroblasts suggests that this molecule controls *Hh* signaling and cellular density within the neonatal lung; and alterations in these processes affect cell cycle exit, and alveolar morphology. Depletion and overexpression of KIF7 in mouse lung epithelial cells demonstrated that this molecule regulates cell proliferation by altering the timing of the G1/S and G2/M transitions. Our work provides the first piece of direct evidence for the role of KIF7 in the regulation of mammalian cell cycle, and suggests that this molecule is required for the maintenance of the alveolar epithelium in postnatal life.

## Results

### KIF7 Is Expressed in the Fetal and Postnatal Lung

**Fig 1:**
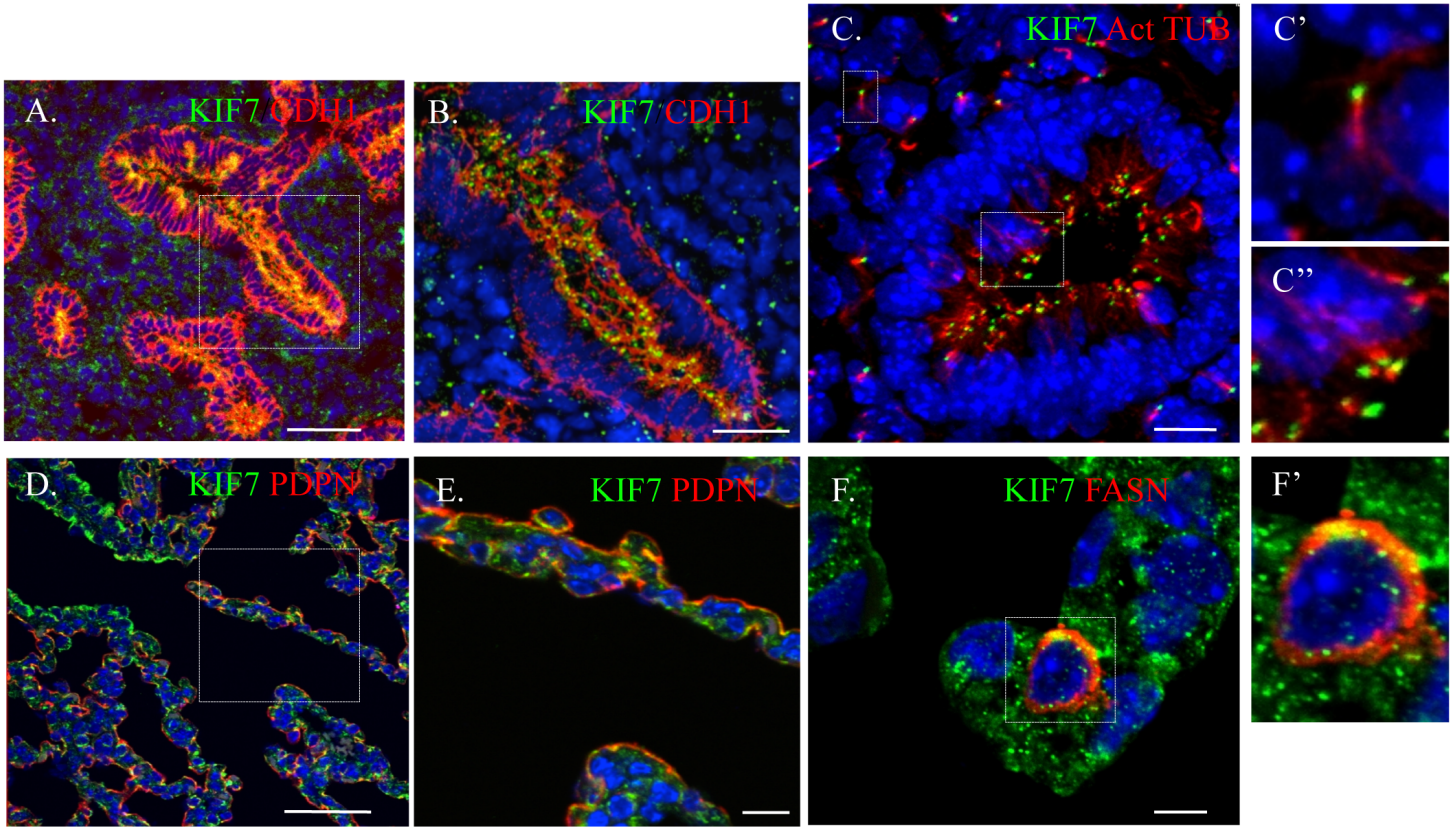
KIF7 is Expressed in the fetal and postnatal lung. (**A.-F’.**) Confocal immunofluorescent staining of KIF7 in the respiratory epithelium. (**A.-B.**) KIF7 co-localization with CDH1 (E-cadherin) at embryonic day E14.5. Scale bars are 50 and 30 microns respectively. **B** is a zoom of the boxed region (**C.**) KIF7 co-localization with acetylated alpha tubulin (a marker of the primary cilium) at E14.5 within the lung epithelium. Scale bar is 10 microns. **C’** and **C”** are zooms from the boxed regions of mesenchymal and epithelial cells in **C**. (**D.-E.**) KIF7 co-localization with PDPN (a marker for the type 1 AEC) at postnatal day 0. **E** is a zoom of the boxed region. Scale bars are 50 and 10 microns respectively. (**F-F’**.) KIF7 co-localization with FASN (a marker for the type 2 AEC) at postnatal day 7. Scale bar is 5 microns. **F’** is a zoom of **F.**

To ascertain *Kif7s* function in the maintenance of the epithelial and mesenchymal cells of the distal lung, we first characterized the expression pattern of KIF7 in the embryonic and postnatal lung using confocal microscopy. We determined that during branching morphogenesis, KIF7 is expressed in both the epithelial and mesenchymal cells of E14.5 fetal lung (Fig 1A and 1B). KIF7 is expressed on the luminal side of CDH1^+^ (E-cadherin) epithelial cells as well as in the mesenchymal cells (Fig 1A and 1B). Higher magnification confocal Z-stacks revealed that KIF7 co-localized specifically with acetylated alpha tubulin, a marker of the primary cilium (Fig 1C) [19]. At E14.5, KIF7 localizes to the tip of primary cilia in both undifferentiated mesenchymal and airway epithelial cells (Fig 1C’ and 1C”). Examination of *Kif7* ^*dd/dd*^ mutant embryos from the same time point revealed an absence of KIF7 protein (S1 Fig), demonstrating that *Kif7* ^*dd/dd*^ is a loss of function of allele. When postnatal alveolar epithelial cells (AECs) were examined, we determined that KIF7 is expressed as punctae in podoplanin^+^ (PDPN) type 1 AECs and fatty acid synthase^+^ (FASN) type 2 AECs [20] (Fig 1D–1F’). These results show that KIF7 is expressed in both primary cilia during embryonic development and in the cytoplasm of secretory epithelial cells in the postnatal lung, suggesting that it may have alternate functions in development and in the maintenance of AECs during postnatal life.

### KIF7 Regulates *Hh* Signaling, Cellular Density, and Cell Cycle Exit in Fibroblasts within the Neonatal Lung

**Fig 2:**
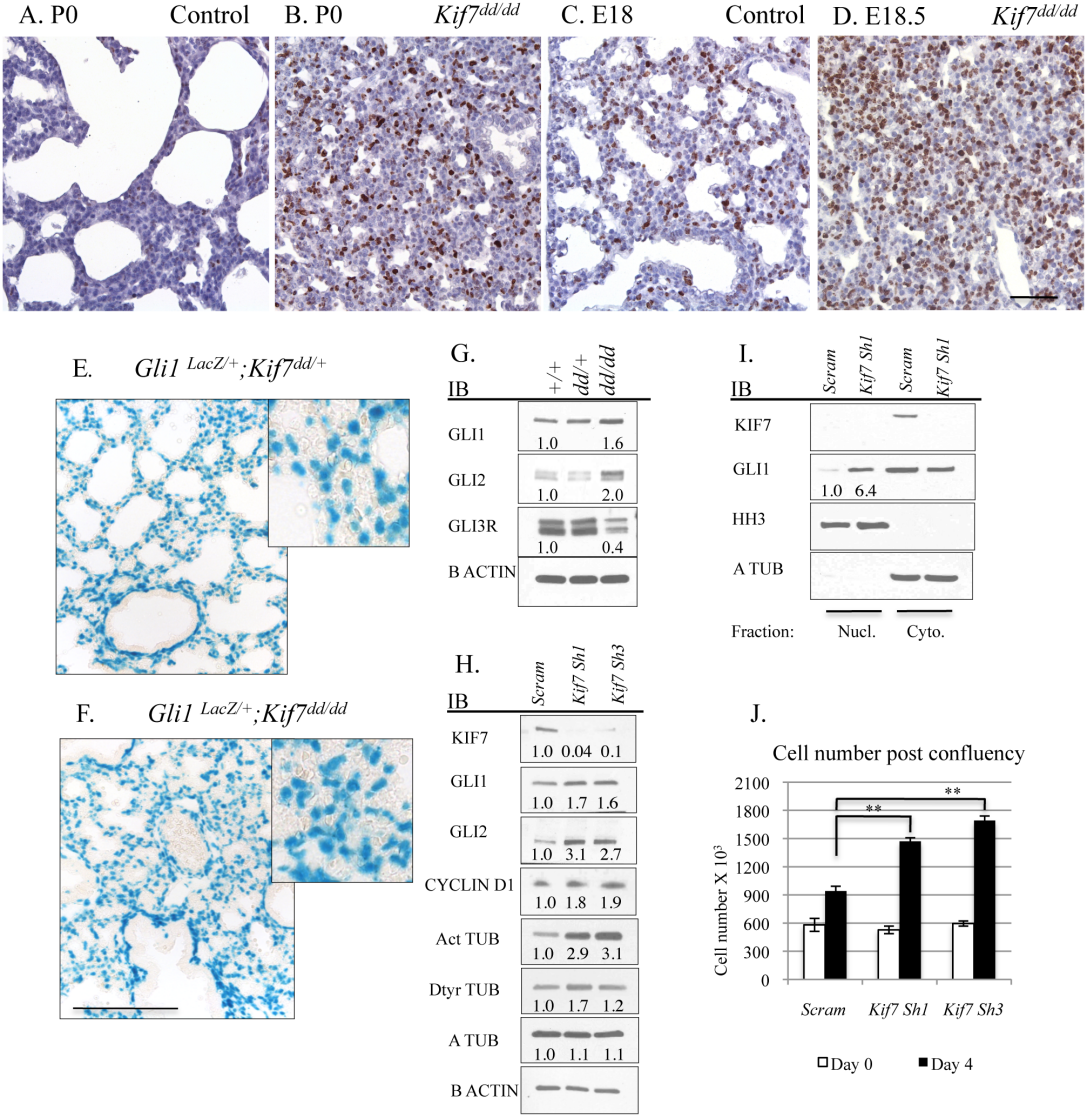
KIF7 Regulates *Hh* Signaling, Cell density, and Cell cycle Exit in Fibroblasts Within the Neonatal Lung. (**A.-D.**) Tissue sections from control and *Kif7* ^*dd/dd*^ mutant embryos were immunostained with the cell proliferation marker Ki67. (**A.+B.**) Sections from postnatal day 0 (P0) control and *Kif7* ^*dd/dd*^ mutant lungs. (**C.+D.**) Representative tissue sections from E18.5 control and *Kif7* ^*dd/dd*^ mutant lungs. Note that P0 *Kif7* ^*dd/dd*^ mutant lungs are denser and have more Ki67^+^ cells than E18.5 control lungs. Scale bar is 50 microns. (**E.-F.**) *Gli1-lacZ Hh* reporter mouse line was crossed into *Kif7* ^*dd/dd*^ mutant mouse line. Images show Beta-galactosidase staining of tissue sections from *Gli1-lacZ;Kif7* ^*dd/+*^ lungs and *Gli1-Lacz; Kif7* ^*dd/dd*^ mutant lungs. Scale bar is 125 microns. (**G.**) Western blot analysis of protein lysates isolated from E18.5 control (+/+), *Kif7* ^*dd/+*^ heterozygous, and *Kif7* ^*dd/dd*^ homozygous mutant lungs. (**H.**) Western blot analysis of protein lysates isolated from postnatal mouse lung fibroblasts (MLFs) infected with control (scram) or *Kif7* gene specific shRNA expressing virus. Cells were infected, grown to confluence, and then serum starved for 3 days to allow for induction of *Hh* signaling. (**I.**) Immunoblots of nuclear fractions isolated from control and *Kif7* shRNA knock down mouse lung fibroblasts. Histone H3 (HH3) was used as nuclear fraction control, and a tubulin as a cytoplasmic control. (**J.**) Quantification of cell density following serum deprivation at 100% cell density. Control cells exit the cell cycle, while *Kif7* depleted MLFs re-enter the cell cycle and divide an additional time. N≥3, * P<0.05, **P<0.01.

We began our investigation by exploring KIF7s function in the mesenchymal cells of the postnatal lung. Because *Kif7* ^*dd/dd*^ mutant embryos die at birth, our analysis of *Kif7* deficient lungs was performed on embryonic day E18.5 and postnatal day 0. At birth, *Kif7* deficient lungs were dense, hypercellular, and contained high numbers of Ki67^+^ proliferating cells (Fig 2A, 2B and S2 Fig). Postnatal day 0 mutant lungs had significantly more Ki67^+^ cells then control lungs at either late gestation (E18.5) or postnatal day 0, suggesting that this dense and hypercellular phenotype was not due solely to developmental delay (Fig 2A–2D). Examination of E18.5 *Kif7* deficient lungs determined that these mutants had reduced elastin deposition adjacent to the respiratory airway. Real time quantitative polymerase chain reaction (RT-qPCR) analysis determined that *Kif7* deficient lungs had decreased elastin (*Eln*) expression, but exhibited normal levels of other transcripts associated with elastin producing mesenchymal cells of the distal lung (*Pdgfra*, *Pdfrb*, *Adrp (Plin2)*, *Pparg*) (S2 Fig) [21,22]. Together these results suggest that the loss of *Kif7* results in dense, hypercellular, and histologically immature lung at birth.

We hypothesized that the hypercellar lung phenotype may be due in part to KIF7s role as a regulator of *Hh* signaling, as the misactivation of the *Hh* signaling pathway by overexpression of *Shh* in distal respiratory epithelial cells has been shown to generate a similar respiratory phenotype at birth, i.e dense hypercellar lungs [23]. To assess this possibility, we first examined the signaling status of the *Hh* pathway using the *Gli1-LacZ* reporter mouse line. *Gli1* is a *Hh* target gene, and *Gli1-LacZ* marks *Hh* responsive fibroblasts within the respiratory airway [24]. Examination of *Kif7* ^*dd/dd*^ *;Gli1-LacZ* mutant lungs showed that the *Hh* pathway was active (at least at the basal level) in both control and mutant lungs (Fig 2E and 2F). To quantify *Hh* pathway activity in *Kif7* ^*dd/dd*^ lungs, western blot analysis was performed for the *Hh* effector molecules GLI1, GLI2, and GLI3R. Immunoblots determined that *Kif7* deficient lungs contained elevated levels of GLI1 and GLI2 proteins (*Hh* pathway activators), as well as a reduction in the expression of GLI3 repressor (Fig 2G)[10]. To determine the cell autonomous role of *Kif7* in regulating the *Hh* pathway in the early postnatal lung, we isolated mouse lung fibroblasts (MLFs) from P0-4 control mice and depleted KIF7 protein using *Kif7* gene specific ShRNAs. As in whole lung extracts, reduced KIF7 expression increased GLI1 and GLI2 protein levels in mouse lung fibroblasts (Fig 2H). To confirm that basal *Hh* signaling was upregulated in *Kif7* depleted mouse lung fibroblasts, we collected nuclear fractions and performed western blots from control and KIF7 depleted cells and observed that GLI1 expression was significantly elevated in the nuclei of KIF7 depleted cells (Fig 2I). These data suggest that KIF7 functions as a negative regulator of *Hh/Gli* signaling in fibroblasts of the perinatal lung, and the mesenchymal cell proliferation phenotypes observed in *Kif7* mutants occurred in part due to altered *Hh* signaling.

**Fig 3:**
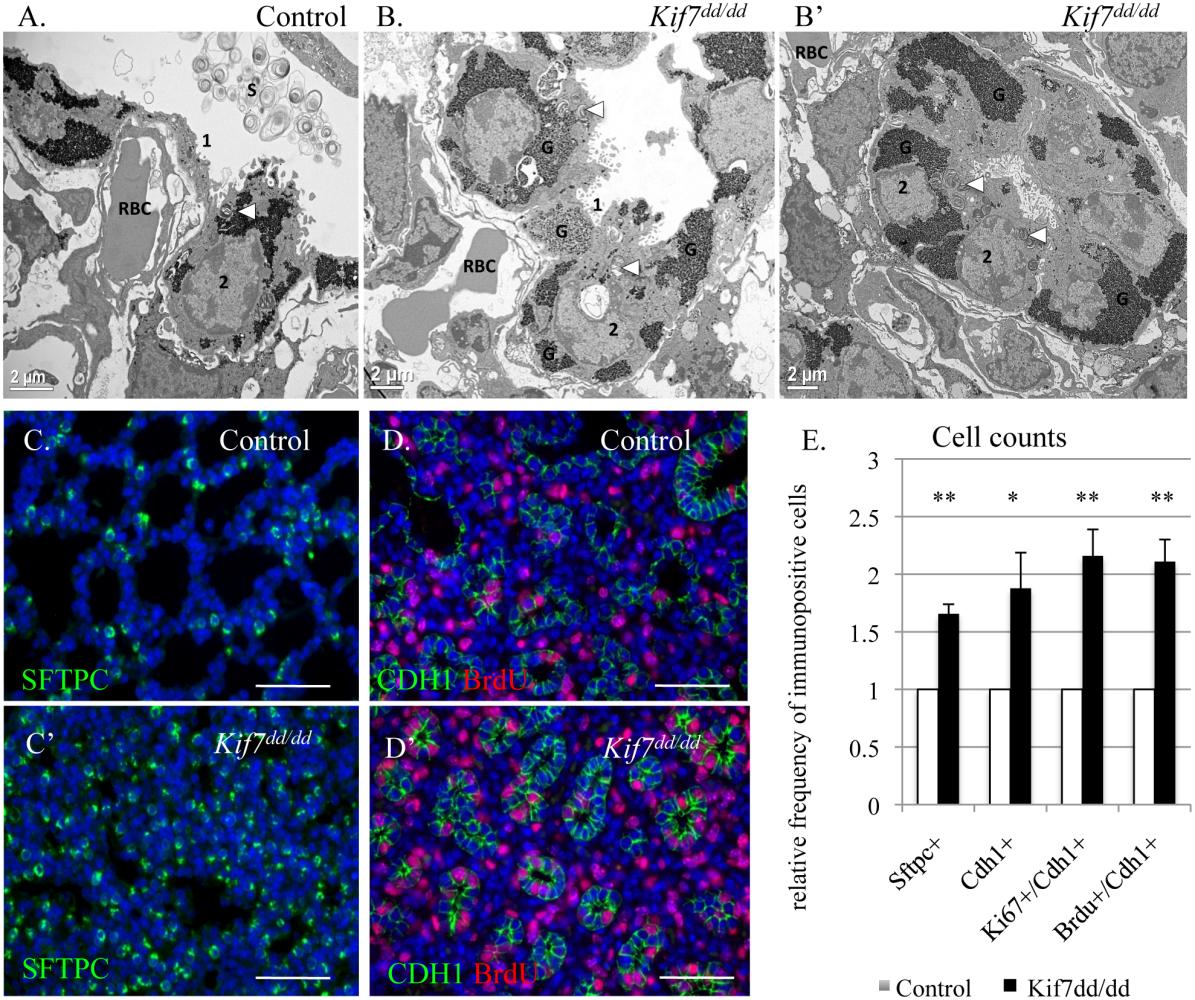
KIF7 is a negative regulator of cell proliferation within the respiratory epithelium. (**A.-B’**) Transmission electron micrographs of E18.5 control and *Kif7* ^*dd/dd*^ mutant respiratory airways. RBC (red blood cell) within the capillary adjacent to a type 1 AEC (1), AEC2 (2), Glycogen (G), Surfactant (S), Arrowheads denote lamellar bodies. (**C.-C’**) Immunofluorescent staining of SFTPC in E18.5 control and *Kif7* ^*dd/dd*^ mutant lungs. (**D.-D’**) Co-immunoflorescent staining of Brdu (red) and CDH1 (green) in E17.5 control and *Kif7* ^*dd/dd*^ mutant lungs. (**E.**) Quantification of relative number of immunopositive cells in control and *Kif7* ^*dd/dd*^ mutant tissue sections. Immunopositive cells were counted and then divided by the total number of cells for control and mutant tissue sections. Control values were set as 1, and mutant values were divided by control values to provide a relative frequency of immunopositive cells per tissue section. At least 4 consecutive tissue sections were counted and averaged for an individual, from at least 4 sets of control and *Kif7* ^*dd/dd*^ mutant lungs. * P<0.05, **P<0.01.

*Hh/Gli* signaling regulates cyclin d expression and overexpression of cyclin d disrupts cell cycle exit and differentiation [25–27]. Since we observed that *Kif7* mutant lungs were dense, histologically immature, and had high levels of Ki67, we examined the expression of cyclin d1 in confluent cultures of *Kif7* depleted MLFs. We determined that inhibition of *Kif7* led to increased expression of cyclin d1, suggesting that the loss of KIF7 may affect cell cycle exit (Fig 2H). As *Kif7* has been implicated in regulating microtubule stability [11,16], we also examined the expression of acetylated alpha tubulin (Act TUB) and detyrosinated alpha tubulin (Dtyr TUB) (2 markers of stable or long-lived microtubules). We observed that postconfluent cultures of *Kif7* depleted MLFs contained higher levels of acetylated alpha tubulin and detyrosinated alpha tubulin (Fig 3H), indicating that KIF7 may negatively regulate microtubule stability in mouse lung fibroblasts.

Next we reasoned that KIF7 functioned to control the density of fibroblasts within the postnatal lung, as *Kif7* mutant lungs appeared dense and hypercellular (Fig 2A–2D). This also seemed probable, as increased expression of GLI1 and GLI2 has been shown to disrupt the growth properties of fibroblasts in culture [29]. To test this, a fibroblast saturation density assay was performed by counting and seeding control and *Kif7* depleted cells at 95% confluency. The following day, cells were counted (day 0), and the remaining wells were serum starved to promote *Hh* signaling. Following 3–4 days of postconfluency in 0.5% serum, *Kif7* depleted MLFs had a higher saturation density compared to control fibroblasts (Fig 2J). Upon confluency, control cells became contact inhibited and did not re-enter the cell cycle while KIF7 deficient cells reentered the cell cycle and completed a second full division (Fig 2J). These data suggest that KIF7 depleted cells may be resistant to cell cycle exit because they reenter the cell cycle and inappropriately divide at confluence. These findings may explain why *Kif7* mutant lungs appear dense, hypercellular, and immature.

### KIF7 Is a Negative Regulator of Cell Proliferation in the Respiratory Epithelium

To determine the role *Kif7* plays in the regulation of cell proliferation and maturation of the respiratory epithelium, the morphology of *Kif7* ^*dd/dd*^ mutant lungs was examined. Transmission electron micrographs (TEM) showed that the alveolar epithelium of *Kif7* ^*dd/dd*^ mutants failed to fully mature, as the AEC 1 cells contained more glycogen and appeared less flat relative to the controls (Fig 3A and 3B) [30]. The type 2 AECs contained lamellar bodies, however we observed decreased surfactant in the respiratory airway. In other regions of the respiratory airway we observed few morphologically normal type 1 AECs, but saw instead rings of alveolar epithelial cells that contained large amounts of glycogen and lamellar bodies indicative of reduced differentiation (Fig 3B’). These data suggest that *Kif7* is required for the maturation of the epithelium of the distal airway.

Immunofluorescent staining for the AEC 2 cell type marker surfactant protein c (SFTPC) revealed that the *Kif7* ^*dd/dd*^ mutant respiratory airway had an increased number of SFTPC^+^ cells (Fig 3C, 3D and 3E). Further immunostaining determined that mutant lungs also contained an increased number of cuboidal E-cadherin (CDH1^+^) respiratory epithelial cells, which was consistent with our TEM analysis (Fig 3B’). We then counted the number of cuboidal CDH1^+^/KI67^+^ cells and determined that E18.5 *Kif7* ^*dd/dd*^ mutant lungs contained increased numbers of Ki67^+^ respiratory epithelial cells suggesting that these cells fail to exit the cell cycle (Fig 3E). To explore the rate of cell proliferation in the respiratory epithelium of *Kif7* ^*dd/dd*^ mutant lungs, the number of BrdU^+^/CDH1^+^ cells were counted in E17 control and *Kif7* mutant lungs (Fig 3D). Analysis of BrdU incorporation revealed that *Kif7* ^*dd/dd*^ deficient lungs had an increased number of epithelial cells in the S phase of the cell cycle relative to controls (Fig 3E). These data indicate that *Kif7* functions as a negative regulator of cell proliferation in the respiratory epithelium, and the loss of *Kif7* disrupts epithelial cell maturation.

### KIF7 Regulates Microtubule Organization, Microtubule Stability, and Cellular Proliferation in Fibroblasts

To further explore the cell biological function of KIF7 in fibroblasts, we examined the phenotypes of mouse embryonic fibroblasts (MEFs) isolated from control and *Kif7* ^*dd/dd*^ (*Kif7* mutant) embryos and KIF7 depleted C3H10T1/2 fibroblasts. Experiments evaluating growth curves and cellular senescence assays showed that *Kif7* mutant MEFs had an increased growth rate and a higher growth potential compared to controls (S3A and S3B Fig). To determine whether *Kif7* mutant MEFs, like lung fibroblasts, develop increased saturation density in culture, control and *Kif7* mutant MEFs were seeded, grown to confluence and then serum starved (0.5% serum) for 15 days. At 15 days, plates were stained with crystal violet, and we observed that *Kif7* mutant MEFs had a higher saturation density than controls (S3C Fig).

Although *Hh* signaling plays a role in the regulation of cell proliferation and entry into S phase [15], we considered whether *Kif7* may be influencing these functions independent from *Hh* independent signaling [16]. Optimal *Hh* signaling requires high cellular densities and cell to cell contact [17]. Therefore, cells were G1 synchronized by serum deprivation at a low cellular density to generate a suboptimal *Hh* signaling environment. Western analysis of protein lysates isolated from control and *Kif7* ^*dd/dd*^ mutant MEFs demonstrated that the loss of *Kif7* did not affect GLI1 (a *Hh* target gene) expression. However, under these conditions we observed that more mutant fibroblasts were immunopositive for cyclin d1 (S3F and S3G Fig), a protein known to be important for the rate of passage from G1 into S phase of the cell cycle [18]. Together, these results suggest that KIF7 is a cell autonomous regulator of cellular proliferation.

**Fig 4:**
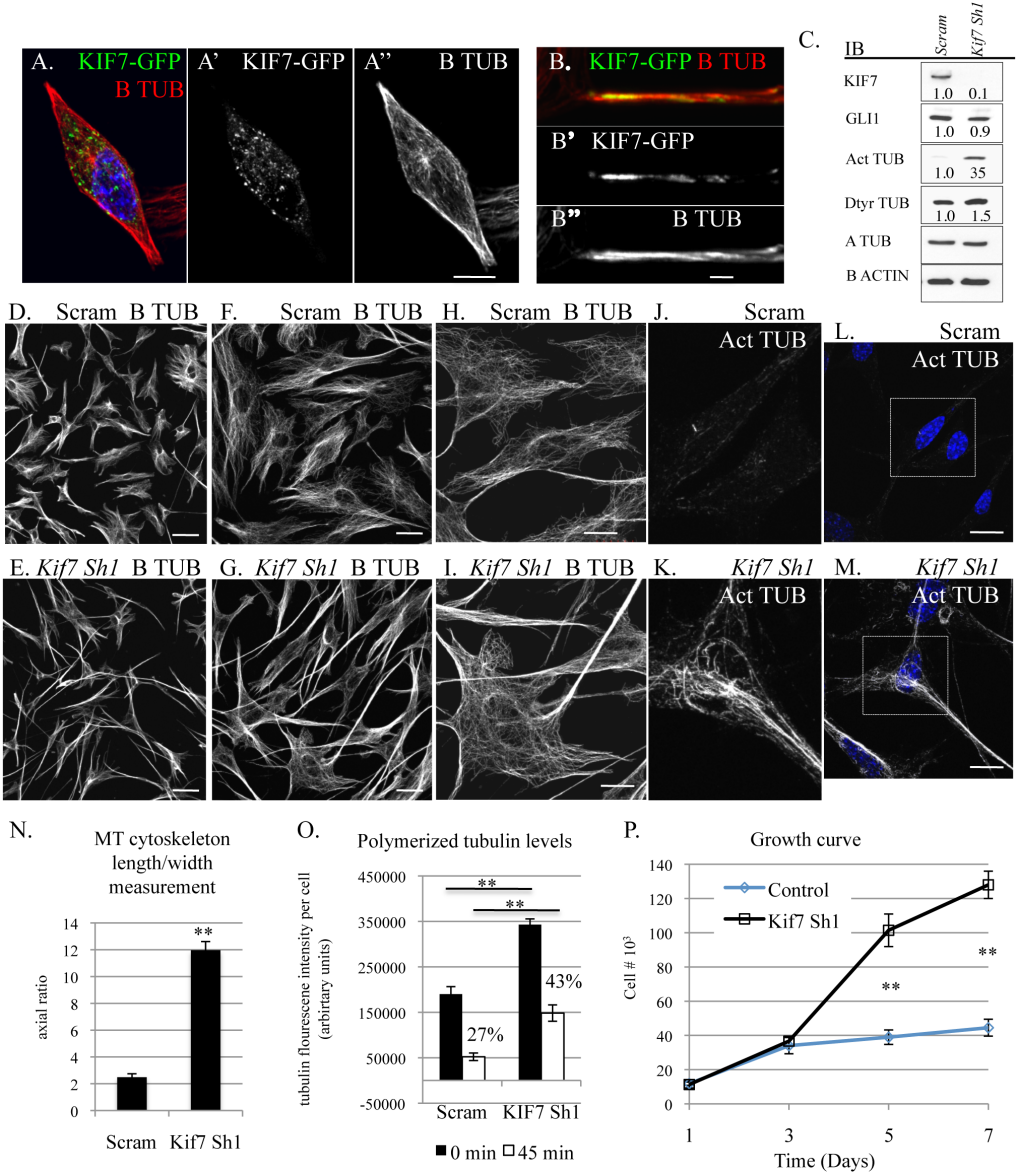
KIF7 regulates microtubule organization, microtubule stability, and cellular proliferation in fibroblasts. (**A.-B”.**) Confocal co-immunofluorescent staining of KIF7-GFP and B tubulin in C3H10T1/2 cells. (**A.-A”**) KIF7 co-localization with B tubulin. **A.** is a merge of A’ and A”. Scale bar is 10 microns. (**B.-B”**) KIF7 co-localization with B tubulin on cytoskeletal microtubules. **B.** is a merge of **B’** and **B”**. Scale bar is 2 microns. (**C.**) Western blots of protein lysates from near confluent asynchronous C3H10T1/2 cells following infection with control (scram) or *Kif7* gene specific shRNA expressing virus. (**D.-I.**) Confocal immunofluorescent staining of B tubulin in scram control and KIF7 depleted C3H10T1/2 cells. Scale bar is 80 microns in **D.+E.**, 40 microns in **F.+G.**, and 20 microns in **H.,I.,L.,M. (J.-M.)** Confocal immunofluorescent staining of acetylated alpha tubulin in scram control and KIF7 depleted C3H10T1/2 cells. **J.**+**K.** are zooms of **L.** and **M.** (**N.**) Average length/width measurement of C3H10T1/2 microtubule cytoskeleton following immunostaining for B tubulin. At least 25 cells were counted from at least 4 independent experiments. (**O.**) Quantification of B tubulin immunofluorescence before and after microtubule depolymerization. KIF7 depleted cells had increased levels of polymerized microtubules at steady state and after incubation on ice for 45 minutes. Fluorescence intensity was measured using ImageJ and divided by the total cell number, to determine average florescence intensity per cell. Control cells contained 27% of their original microtubule polymer mass following incubation on ice, while KIF7 depleted cells contained 43% of their original polymer mass under the same conditions. Approximately 25 cells were counted from 5 independent fields to generate an average, and the experiment was repeated 3–5 times. (**P.**) Growth curve of control (Scram) and KIF7 depleted MLE15 cells. N≥3, * P<0.05, **P<0.01.

Under standard culture conditions, we observed that mouse embryonic fibroblasts showed heterogeneity in microtubule organization; therefore we chose to explore KIF7s function in the monoclonal fibroblast cell line, C3H10T1/2. We chose these cells as they were derived from mouse embryos and have been shown to be sensitive to contact inhibition of cell proliferation [31]. We hypothesized that depletion of KIF7 from a monoclonal cell line would generate a uniform phenotype allowing for a more detailed analysis. When we examined the expression of KIF7 (using a KIF7-GFP fusion construct) in C3H10T1/2 cells, we determined that KIF7 was expressed throughout the cytoplasm and that it co-localized with cytoskeletal microtubules (Fig 4A and 4B). We then depleted KIF7 from C3H10T1/2 cells and examined KIF7s role in the regulation of microtubule organization, cytoskeletal maintenance, and cellular proliferation. Western analysis of asynchronous C3H10T1/2 cells revealed that loss of KIF7 led to a dramatic increase in both acetylated and detyrosinated alpha tubulin levels without affecting the expression of *Hh* target GLI1. These findings suggest that KIF7 functions to regulate microtubule stability in C3H10T1/2 cells independent of its role in regulating *Hh* signaling.

We next used confocal microscopy to evaluate KIF7s influence on microtubule organization in C3H10T1/2 cells. Loss of KIF7 dramatically disrupted cell morphology and microtubule organization as KIF7 depleted C3H10T1/2 cells became increasingly polarized (Fig 4D–4I). We observed that microtubules within KIF7 depleted cells were simplified and extremely elongated relative to control cells (Fig 4F–4I). We quantified the cytoskeletal phenotypes by calculating the average length/width ratio of the microtubule cytoskeleton in control and KIF7 depleted cells. Loss of KIF7 led to a 4 fold increase in the average length/width ratio of the microtubule cytoskeleton (Fig 4N). When we examined acetylated alpha tubulin localization, we determined that this posttranslational modification had accumulated throughout cytoskeleton of KIF7 depleted cells, suggesting that cytoskeletal microtubules were under processed. These results show that KIF7 functions to regulate microtubule processing and cytoskeletal organization in fibroblasts, and that these functions occur independent of Hh/ciliary signaling.

As we identified profound changes in microtubule organization and increased expression of acetylated and detyrosinated alpha tubulin, we hypothesized that KIF7 functions to regulate microtubule dynamics within the cytoskeleton of fibroblasts, similar to what has been described in human retinal pigment epithelial (RPE) cells [16]. We used a microscopy based quantitative immunofluorescent assay to determine the amount of polymerized tubulin before and after microtubule depolymerization on ice to determine if KIF7 controls cytoskeletal microtubule stability in mouse fibroblasts [16,32]. When we quantified B tubulin fluoresce intensity following methanol stabilization of tubulin polymers, we determined that KIF7 depleted cells had increased levels of polymerized tubulin (Fig 4O and S5 Fig). Similar to a previous report, we found that microtubules in KIF7 depleted cells were resistant to depolymerization on ice [16]. After 45 minutes at 4 degrees, control cells contained 27% of their original polymerized microtubule polymer mass, while KIF7 depleted cells contained 43% of their original microtubule polymer mass (Fig 4O). These results suggest that KIF7 functions to regulate microtubule organization and microtubule stability within mouse fibroblasts.

Next, we examined growth curves to determine how the loss of KIF7 affected cell proliferation in C3H10T1/2 cells. We found that KIF7 depleted cells had a significant growth advantage under high cellular densities, a phenotype similar to what we observed in both MEFs and mouse lung fibroblasts (Fig 4P). We measured cell size by determining the surface area of control and KIF7 depleted cells after co-immunofluorescent staining for B tubulin and F actin (S4 Fig). We did not detect a statistically significant change in the area of KIF7 depleted cells, suggesting that the increased saturation density does not occur secondary to changes in cell size (S4 Fig). Instead, we hypothesize that altered microtubule dynamics may contribute to a reduction in contact inhibition of cell proliferation *in vitro* and the phenotypes observed in *Kif7* ^*dd/dd*^ mutant lungs.

### KIF7 Regulates Cell Proliferation and Entry into S Phase in Mouse Lung Epithelial Cells

Our analysis of the *Kif7* mutant distal respiratory epithelial phenotype suggests that the loss this gene led to increased cell proliferation and disruption of alveolar epithelial cell (AEC) maturation (Fig 3). While examining the alveolar epithelium using transmission electron microscopy (TEM), we were unable to identify primary cilia on type 2 AECs. A literature review of previously published EM data also failed to identify primary cilia on AECs, suggesting that mature AECs may not have primary cilia [33,34]. Confocal co-immunofluorescent staining for markers of primary cilia and AECs in tissue sections of E18.5 lungs further suggested that AECs are non-ciliated cells (S5 Fig).

Next, we used an *in vitro* mouse lung epithelial cell line (MLE15) to further explore the function of *Kif7* in AECs. MLE15 cells are immortalized distal respiratory epithelial cells that express markers of the AEC 2 lineage such as surfactant protein b and c [35]. When we characterized these cells, we determined that they were cuboidal epithelial cells, and unlike mouse lung fibroblasts (MLFs), they failed to produce ciliary like structures following cell synchronization after serum withdrawal. Instead, acetylated alpha tubulin (a marker of primary cilia) partially co-localizes with the centrosome marker gamma tubulin (S5 Fig).

We then tested if mouse lung epithelial cells were capable of responding to *Hh* signaling. We treated confluent, serum starved MLE15 cells and mouse lung fibroblasts with the Hh/Smo agonist SAG, and examined the expression of *Gli1* (S5 Fig) [36]. We determined that lung fibroblasts were Hh responsive, since SAG treatment led to a 100 fold induction of the *Hh* target gene *Gli1*. Mouse lung epithelial cells were not responsive to Hh pathway stimulation, as treatment with SAG did not induce *Gli1* expression. We suspect that MLE15 cells were unable to respond to Hh stimulation because they lacked primary cilia. These results are consistent with a recent report showing that *Gli1-lacZ* (which marks *Hh* responsive cells) is expressed in fibroblasts but not in the epithelial cells of the distal lung [24]. These experiments suggest that MLE15 cells are an ideal model for isolating and understanding the cellular functions of KIF7 without the complexity of signaling within the primary cilium.

**Fig 5:**
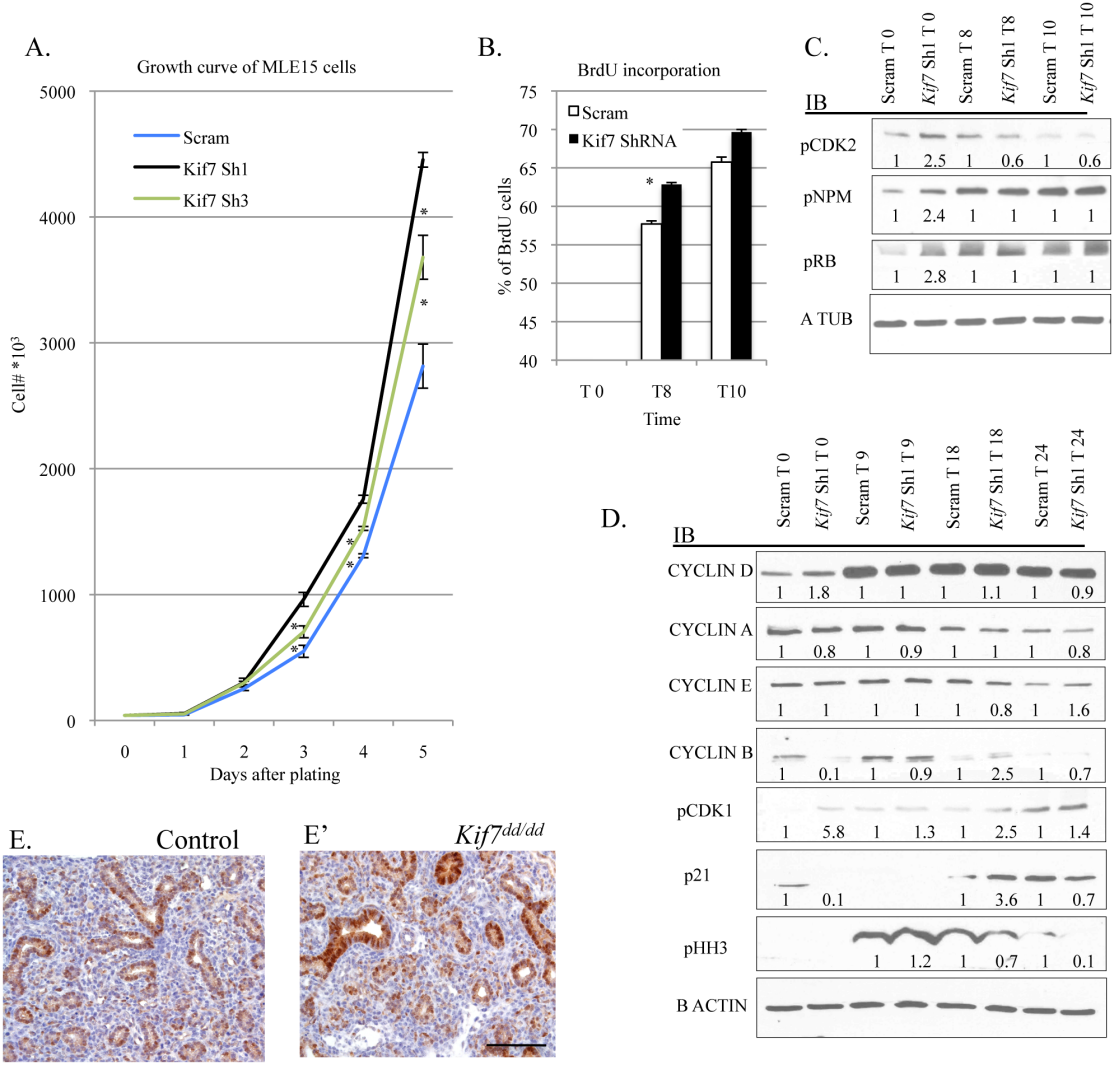
KIF7 Regulates cell proliferation and entry into S phase in mouse lung epithelial cells. (**A.**) Growth curve of control (Scram) and KIF7 depleted MLE15 cells. (**B.**) BrdU incorporation was measured by flow cytometry after release from G1 synchronization for control and KIF7 depleted cells. The analysis was performed using the BD BrdU-FitC kit and FlowJo software. See S6 Fig for representative flow charts. (**C.**) Western blots of protein lysates from time course of serum starved G1 synchronized MLE15 cells (Time 0, and 8 or 10 hours after the re-addition of serum continuing media). (**D.**) Western blots of protein lysates from time course of G1 synchronized cells (Time 0) and following re-addition of serum containing media. (**E.+E’**) Immunostained tissue sections of control and *Kif7* mutant lungs. Cyclin d1 expression (brown) appears elevated in the *Kif7* mutant respiratory epithelium. Scale bar is 100 microns. N≥3, * P<0.05, **P<0.01.

To better understand the function of KIF7 in the respiratory epithelium, we depleted this protein in MLE15 cells by infection with lentivirus expressing one of two independent *Kif7 ShRNA* constructs, or scrambled (scram) control virus. The *Kif7* ShRNA constructs produced a 60–80% reduction in KIF7 expression relative to the scram control (S6 Fig). To test whether KIF7 functioned to regulate cell proliferation *in vitro*, control and *Kif7* depleted cells were counted and plated for growth analysis. Depletion of KIF7 increased the rate of cell proliferation compared to the controls (Fig 5A). Based on these data, we hypothesized that loss of KIF7 affects the timing of the G1/S transition. To ascertain whether loss of KIF7 alters entry into S phase, MLE15 cells were synchronized at the restriction point in G1 by serum deprivation (cell synchronization was confirmed by flow cytometry). Flow cytometric analysis of BrdU incorporation following release from the restriction point in G1, showed that KIF7 depleted cells moved into S phase (and incorporated BrdU) faster than controls (Fig 5B and S6 Fig).

Decreased expression of *KIF7* has been associated with abnormal centrosome duplication in human retinal pigment epithelial (RPE) cells [16]. As KIF7 depleted mouse lung epithelial cells entered into S phase precociously, and centrosome duplication occurs in S phase, we considered the possibility that this centrosome duplication phenotype was due to disruption of signaling at the G1/S checkpoint. Cyclin e and cyclin-dependent kinase 2 (CDK2) regulate entry into S phase at the G1/S checkpoint by phosphorylation and inactivation of Retinoblastoma protein (RB). CDK2 is also believed to regulate centrosome duplication by phosphorylation of nucleophosmin (NPM) [8,9]. To determine whether CDK2 activity and centrosome signaling were perturbed in KIF7 depleted MLE15 cells, western blots were performed on lysates from G1 arrested cells before and after the readdition of serum. KIF7 depleted cells had increased CDK2 activity (seen by CDK2 phosphorylation), as well as increased phosphorylation of RB and NPM (Fig 5C). These results suggest that abnormal centrosome duplication phenotype of KIF7 depleted RPE cells may be due to increased CDK2 activity.

To identify why loss of KIF7 altered entry into S phase, we examined the expression of the cyclins and major cell cycle inhibitors. Control and KIF7 depleted cells were synchronized by serum starvation in G1, and western blots were performed on lysates before and after the readdition of serum. At time 0, KIF7 depleted cells had higher levels of cyclin d1 and pCDK1 and reduced levels of cyclin b and the cell cycle inhibitor p21 at the restriction point. Following the readdition of serum, cyclin b accumulated in a normal fashion, and we observed an accelerated accumulation of pCDK1, p21, and phospho histone H3 (a marker for the G2 and M phases of the cell cycle) (Fig 5D) [37]. Cyclin d1 expression was then evaluated *in vivo*, and we determined that cyclin d1 expression appeared elevated in the epithelium of *Kif7* mutant lungs, relative to controls (Fig 5E and 5E’). These results suggest that KIF7 regulates the rate of passage from the restriction point into S phase, and this occurs in part through increased expression of cyclin d1, increased CDK2 activity, increased RB phosphorylation, and decreased p21 levels in the G1 phase of the cell cycle.

### KIF7 Regulates Mitotic Exit in Mouse Lung Epithelial Cells

**Fig 6:**
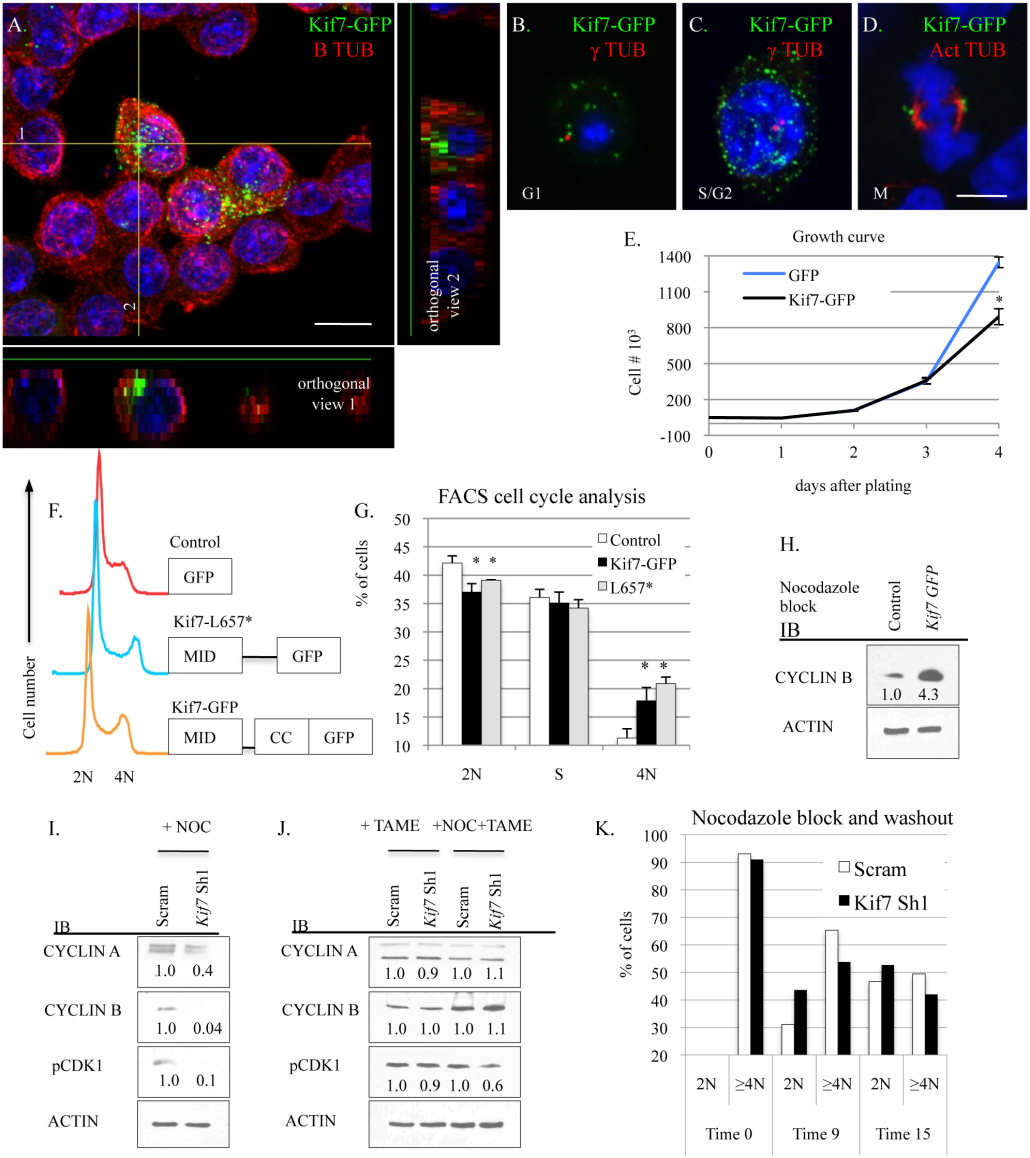
KIF7 regulates mitotic exit in mouse lung epithelial cells. (**A.-D.**) Confocal immunofluorescent analysis of KIF7-GFP expression in different stages of the MLE15 cell cycle. **A.** G1 synchronized MLE15 cells expressing KIF7-GFP. **B.** A MLE15 cell in G1 with an unduplicated centrosome. **C**. A MLE15 cell in S/G2 with duplicated centrosomes, **D**. A MLE15 cell in mitosis. (**A.**) Confocal Z-stack of KIF7-GFP and B tubulin. Note that KIF7 localizes predominately to one side of the cell. Scale bar is 10 microns. (**B.+C.**) Co-immunofluorescent staining of KIF7-GFP with gamma (γ) tubulin, a marker for the centrosome. (**D.**) Co-immunofluorescent staining of KIF7-GFP colocalization with acetylated alpha tubulin, a marker of the spindle apparatus. Scale bar is 5 microns. (**E.**) Growth curve for KIF7-GFP overexpression in MLE15 cells. Cell proliferation was examined in GFP expressing and KIF7-GFP expressing MLE15 cells. (**F.**) Cell cycle profile for GFP, KIF7-GFP, and KIF7 L657*-GFP mutant expressing cells. (**G.**) FACs cell cycle analysis of *Kif7* overexpressing MLE15 cells. (**H.**) Western blots of protein lysates from GFP expressing (control) and Kif7-GFP expressing cells synchronized at the G2/M cell cycle checkpoint by nocodazole treatment. (**I.-J.**) Western blots of protein lysates from scram control and KIF7 depleted cells synchronized at the G2/M checkpoint with treatment with (NOC) nocodazole, TAME HCL (an anaphase promoting complex inhibitor), or nocodazole and TAME together. (**K.**) Flow cytometric analysis of DNA content following nocadazole washout for control and KIF7 depleted cells. KIF7 depleted cells routinely move from 4N to 2N faster than Scram controls. N≥3, * P<0.05, **P<0.01.

To improve our understanding of how KIF7 regulates cell cycle progression in respiratory epithelial cells, we examined the expression of KIF7 throughout the cell cycle. Immunostaining of G1 synchronized KIF7-GFP expressing cells revealed that KIF7 is expressed throughout the B tubulin^+^ cytoskeleton of MLE15 cells. When we examined orthogonal views of confocal Z-stacks, we determined that KIF7-GFP was concentrated along one side of MLE15 cells (Fig 6A). We hypothesized that this structure was the microtubule tubule-organizing center, or centrosome. Further immunostaining revealed that in asynchronous cells, KIF7-GFP localizes to regions surrounding the gamma tubulin^+^ centrosome in G1, S, and G2 phases of the cell cycle, confirming our hypothesis (Fig 6B and 6C). We also observed that KIF7-GFP localized to the acetylated alpha tubulin^+^ spindle pole during the M phase of the MLE15 cell cycle (Fig 6D). To evaluate whether increased KIF7 expression was sufficient to alter the rate of MLE15 cell proliferation, KIF7-GFP stable expressing MLE15 cells were generated and a growth curve was performed. KIF7-GFP expressing cells grew significantly slower than GFP control cells (Fig 6E). To determine which domains of KIF7 were important for regulating cell proliferation, full length KIF7 containing the microtubule interacting domain, the coil-coiled domain (KIF7-GFP), and a truncated KIF7 containing only the microtubule interacting domain (KIF7-L657*) were over-expressed in MLE15 cells. Cell cycle analysis of KIF7-GFP, KIF7-L657*, and GFP expressing cells demonstrated that overexpression of KIF7-GFP and KIF7-L657* generated a partial G2/M cell cycle arrest relative to the GFP control cells (Fig 6F and 6G), indicating that the KIF7 microtubule interacting domain is sufficient to regulate cell proliferation in MLE15 cells.

We were curious to know why overexpression of KIF7 disrupted cell cycle progression, so we examined the cell signaling phenotypes of KIF7-GFP expressing cells following nocodazole synchronization at the G2/M checkpoint. Immunoblots of lysates from nocodazole arrested GFP and KIF7-GFP expressing cells determined that KIF7-GFP overexpressing cells contained elevated levels of cyclin B at the G2/M checkpoint (Fig 6H). Conversely, immunoblots of nocodazole synchronized KIF7 depleted cells showed that the inhibition of *Kif7* expression led to reduced expression of cyclin a, cyclin b, and active CDK1 at the G2/M checkpoint (Fig 6I). To determine if these expression changes occurred due to misactivation of the anaphase promoting complex (APC), TAME-HCL (an APC inhibitor) was used to treat control and KIF7 depleted synchronized cells at the G2/M checkpoint. Inhibition of APC activity restored cyclin a, cyclin b, and active CDK1 levels in KIF7 depleted cells, suggesting that the timing of the anaphase-promoting complex was disrupted by inactivation of KIF7 in MLE15 cells (Fig 6J) [38]. We hypothesized that decreased cyclin b and active CDK1 may disrupt the timing of mitosis, as these molecules need to be degraded for mitotic exit [7]. To test this, control and KIF7 depleted cells were synchronized and released from the G2/M checkpoint, and cellular DNA content was monitored by flow cytometry. KIF7 depleted MLE15 cells repeatedly moved from 4N back to 2N faster than control cells (Fig 6K). These data suggest that KIF7 is necessary and sufficient to regulate cell proliferation in MLE15 cells. Additionally, as over expression of KIF7 resulted in a partial G2/M cell cycle arrest, and KIF7 depleted cells exited mitosis faster than controls, we conclude that KIF7 is necessary for normal exit from mitosis in mouse lung epithelial cells.

### KIF7 Regulates Microtubule Stability in Respiratory Epithelial Cells

**Fig 7:**
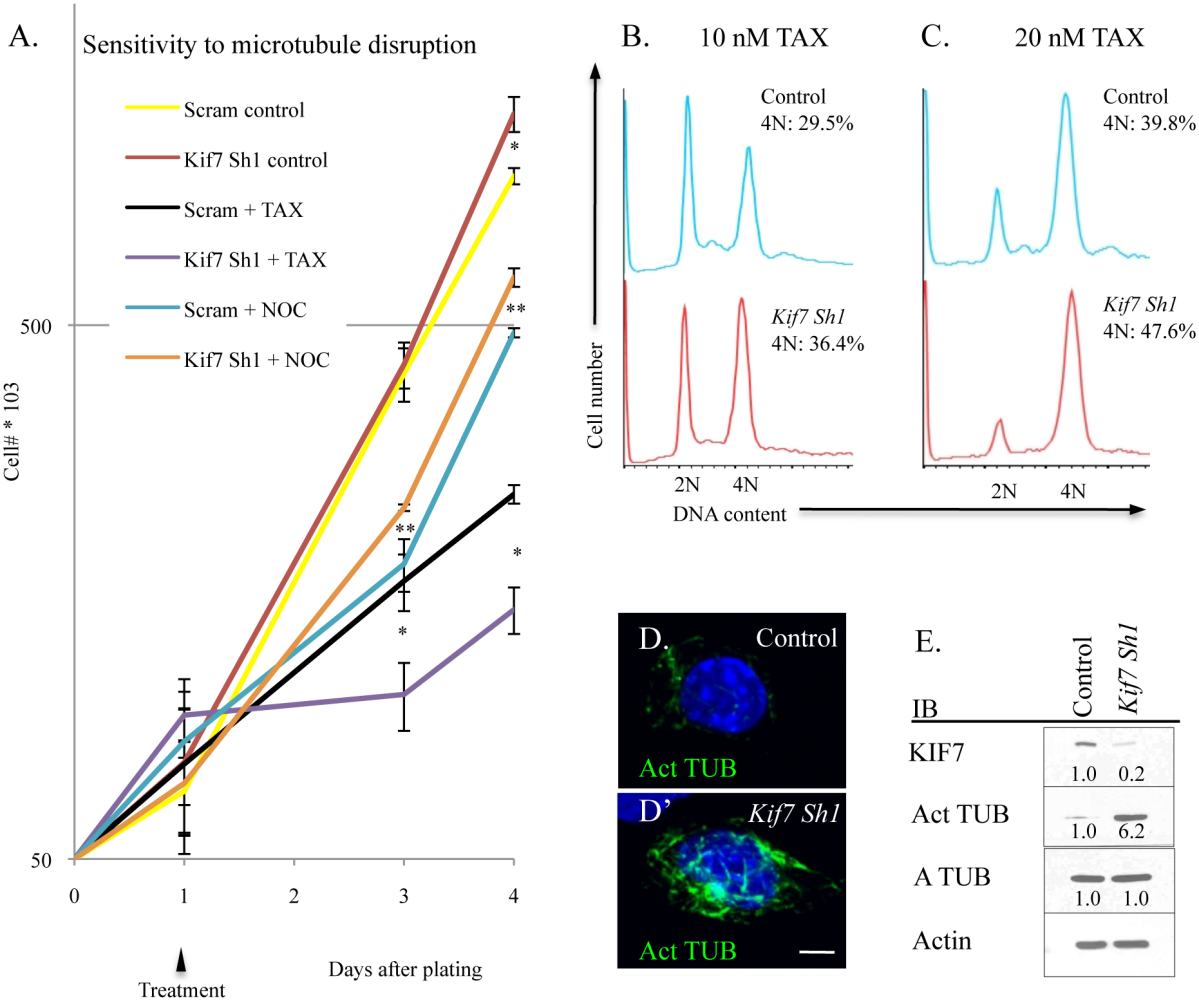
KIF7 regulates microtubule mstability in mouse lung epithelial (MLE15) cells. (**A.**) Growth curves of control (Scram) and KIF7 depleted cells following treatment and washout with the microtubule stabilizing drug, paclitaxel (TAX), and the microtubule depolymerizing drug, nocodazole (NOC). Cells were counted and plated, and then treated 24 hours later (Day 1). 18 hours later (Day 2), cells were washed 2 times in PBS, and then incubated with complete media. (**B.-C.**) Flow cytometric analysis of DNA content after overnight incubation with paclitaxel. (**D.+D’**) Confocal Z stacks of immunofluorescent staining of acetylated alpha tubulin in control and KIF7 depleted MLE15 cells. (**E.**) Western blot analysis of high passage asynchronous MLE15 cells. N≥3, * P<0.05, **P<0.01.

KIF7 has previously been shown to regulate microtubule stability in non-dividing cells; therefore we explored how KIF7 depletion affects microtubule stability in proliferating mouse lung epithelial cells [11,28]. To test this, MLE15 cell growth was evaluated after treatment with paclitaxel (to stabilize microtubules) and with nocodazole (to prevent microtubule polymerization). Depletion of KIF7 (*Kif7* Sh1) led to increased sensitivity to paclitaxel, and decreased sensitivity to nocodazole administration (Fig 7A). To ascertain why KIF7 depleted cells were sensitive to microtubule stabilization, the cell cycle status of MLE15 cells was examined by flow cytometry following a 24 hour treatment with paclitaxel. DNA content analysis determined that KIF7 depletion produced more cells with a DNA content of 4N following paclitaxel treatment, relative to control cells (Fig 7B and 7C). Confocal and western analysis of high passage KIF7 depleted cells revealed that loss of KIF7 led to widespread microtubule acetylation (Fig 7D and 7E). These results suggest that KIF7 depleted mouse lung epithelial cells have increased microtubule stability, which reduces the threshold required for induction of the spindle, assemble checkpoint. Taken together, these findings suggest that KIF7 maintains the microtubule cytoskeleton in proliferating mouse lung epithelial cells.

### Disruption of Microtubule Dynamics Alters the Expression and Activity of Numerous Components of Cell Cycle Machinery

Our work demonstrated that KIF7 (KIF7-GFP) was associated with the centrosome and the spindle pole in nonciliated respiratory epithelial cells (Fig 6). As several proteins misexpressed in KIF7 depleted cells are associated with the centrosome (CDK2, cyclin b, p21)[5,39–41], we hypothesized that KIF7 may interact with these proteins to regulate their stability. To test this, co-immunoprecipitation experiments were performed in KIF7-GFP expressing MLE15 cells. These experiments yielded no evidence for an interaction between KIF7 and cyclin b, cyclin e, pNPM, pCDK2, p21, or either of the Anaphase Promoting Complex (APC) complex cofactors, FZR1 (CDH1) or CDC20 (p55) (S7 Fig) [42–45].

**Fig 8:**
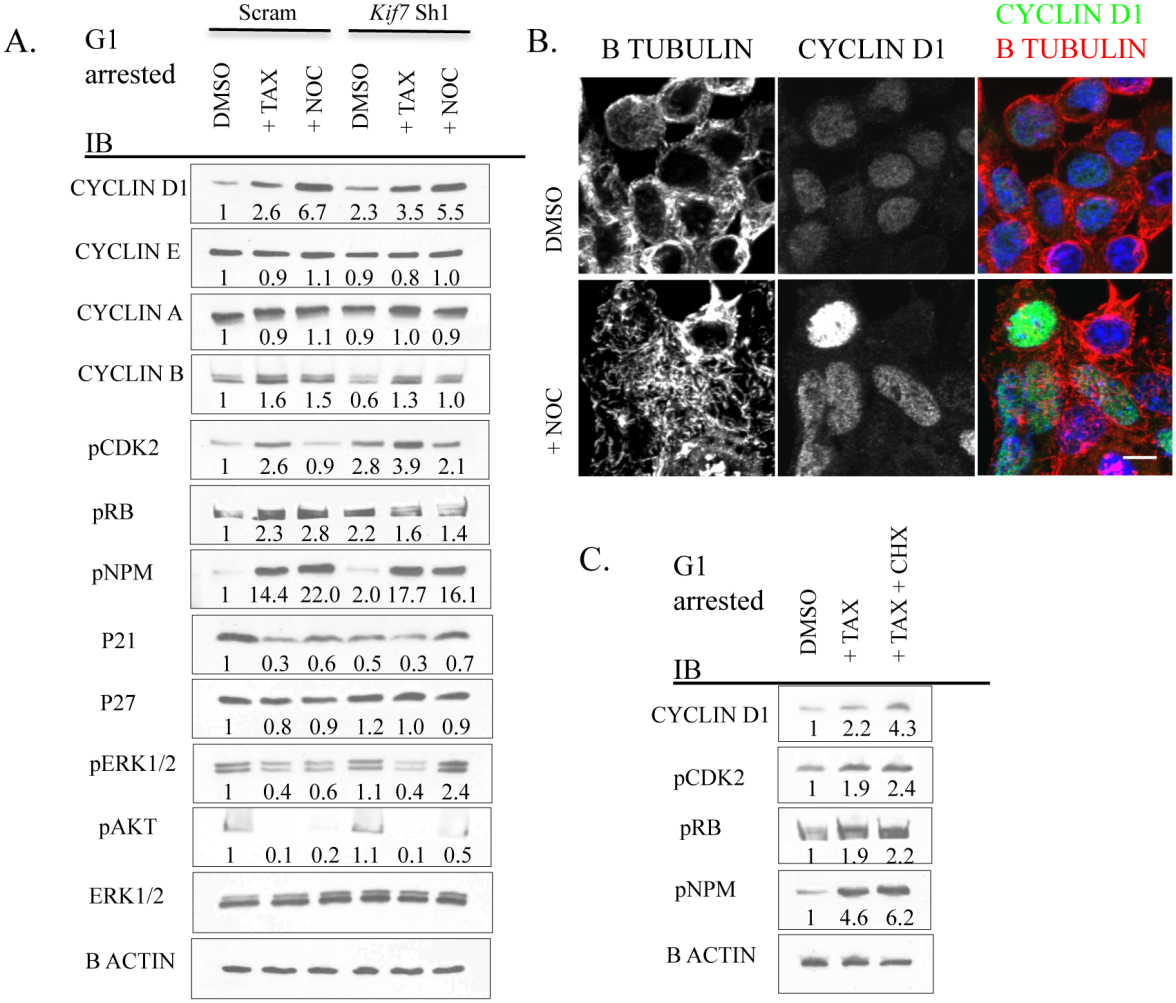
Disruption of microtubule dynamics alters the expression and activity of numerous components of cell cycle machinery. (**A.**) Representative immunoblots of Scram control and KIF7 depleted G1 synchronized MLE15 cells following a 6–8 hour treatment with DMSO (control), paclitaxel (TAX), or nocodazole (NOC). (**B.**) Immunofluorescent confocal images of MLE15 cells following treatment as in **A.** (**C.**) Immunoblots of MLE15 cells treated with DMSO and H_2_O (control), paclitaxel, or paclitaxel and cycloheximide (CHX) as in **A**.

We then hypothesized that disruption of microtubule dynamics in G1 was altering the expression of cell cycle machinery proteins, as microtubules regulate mitotic protein degradation during mitosis [37]. To test this, control and KIF7 depleted MLE15 cells were synchronized in G1 by serum deprivation and then treated with paclitaxel or nocodazole for 6–8 hours (Fig 8A). Western blot analyses determined that stabilizing microtubules in control cells (paclitaxel treated) led to increased expression of cyclin d1, cyclin b, pCDK2, pRB, and pNPM with decreased levels of p21. Depolymerization of microtubules by nocodazole administration also led to increased cyclin d1, cyclin b, pNPM, and decreased p21 levels. KIF7 depleted cells responded similarly with the exception that nocodazole treatment restored cyclin b levels to normal, reduced pCDK2 and pRB, and partially restored p21 levels to normal, suggesting that increased microtubule stability contributes to the phenotypes observed in KIF7 depleted cells (Fig 8A).

Growth factor signaling was then examined by western analysis of phosphorylated extracellular signal-regulated kinase (pERK1/2) and phosphorylated protein kinase B (pAKT) as these pathways have been implicated in regulating the expression and activity of the cell cycle machinery in G1 [6,46]. No change was observed in the expression of pERK1/2 or pAKT in KIF7 depleted cells relative to control cells. Examination of cyclin d1 localization following microtubule depolymerization revealed that disruption of microtubules promoted the accumulation of this protein within the nucleus (Fig 8B). To differentiate whether microtubule disruption alters the expression of these factors at the mRNA or protein level, MLE15 cells were treated with paclitaxel or paclitaxel plus cycloheximide to simultaneously stabilize microtubules and inhibit protein synthesis. As shown in Fig 8C, addition of paclitaxel led to increased post-transcriptional expression of cyclin d1, pCDK2, pNPM, and pRB. This suggests that microtubule dynamics affect the expression of these cell cycle molecules in G1, and this occurs in part through a post-transcriptional mechanism. Together, these data demonstrate that increased microtubule stability in G1 is sufficient to increase the expression and activity of several key cell cycle regulators which were perturbed in KIF7 depleted cells. Most importantly, as treatment of KIF7 depleted cells with nocodazole partially restored the expression of pCDK2, p21, and cyclin b, this shows that the cell cycle phenotypes observed were due at least in part to altered microtubule dynamics.

## Discussion

In our study, we provide evidence for a model in which KIF7 and microtubules regulate cell proliferation by controlling the expression of several key molecules in the G1 phase of the cell cycle. We observed that loss of KIF7 increased the rate of cell proliferation, the saturation density, and the growth potential of mouse embryonic fibroblasts (S3 Fig). Analysis of *Kif7* ^*dd/dd*^ mutant lungs determined that the KIF7 was necessary for the maturation of both the mesenchymal cells and the type 1 and type 2 alveolar epithelial cells of the respiratory airway and (Figs 2, 3 and S2 Fig). Our work suggests that KIF7 regulates cell density within the neonatal lung in part through controlling *Hh/Gli* signaling, cyclin d1 expression, and microtubule mediated contact inhibition of cell proliferation (Figs 3 and 4). Previous work has shown that ectopic expression of cyclin d1 is sufficient to disrupt differentiation, possibly through alterations in cell cycle exit [26,27]. It is therefore likely that the developmental phenotypes present in *Kif7* mouse mutants may be due to a shift in the balance between cell proliferation and cell cycle exit, similar to what has been observed for mutations in other cilia/microtubule associated proteins [13].

We observed that depletion of KIF7 from MLE15 cells (which to our knowledge lack primary cilia), was associated with increased microtubule stability and increased cell proliferation demonstrating that this protein regulates these processes independent of its role as a regulator of ciliary architecture [11]. We determined that KIF7 depletion from MLE15 cells accelerates entry into S phase in part through increased cyclin d1 expression, increased CDK2 activity, and reduced expression of the cell cycle inhibitor p21 (Fig 5). Previous studies have established that overexpression of cyclin d1 reduces the length of G1 and accelerates the rate of the G1/S transition [18]. Similarly, we determined that depletion of KIF7 from MLE15 cells increased the rate of the G1/S transition consistent with the effect of increased cyclin d1 expression in the G1 phase of the cell cycle (Fig 5).

Cyclins and cyclin dependent kinases have numerous functions including regulation of the G1/S transition, centrosome duplication, DNA replication, and exit from mitosis [5,7]. Our experiments suggest that loss of *Kif7* accelerates the timing of cyclin/CDK activity in G1 as cyclin d1, pCDK1, and pCDK2 are prematurely up regulated. We hypothesize that the timing of cyclin/CDK activity is accelerated throughout the entire cell cycle in KIF7 depleted cells as we observed that cyclin b and pCDK1 were prematurely degraded by the anaphase promoting complex (APC) at the G2/M checkpoint, a molecular phenotype that could be rescued by treatment with the APC inhibitor, TAME (Fig 6). Previous studies have shown that CDK1 also functions to phosphorylate and activate the APC complex [43]. We observed that CDK1 phosphorylation occurred prematurely in KIF7 depleted MLE15 cells, which may imply that acceleration of the timing of cyclin/CDK activity in G1 leads to early activation of APC, thereby increasing the rate of exit from mitosis.

Transient suppression of *KIF7* expression has previously been shown to promote centrosome duplication in G1 arrested human retinal pigment epithelial (RPE) cells [16]. This phenotype intrigued us because we observed that KIF7 depleted MLE15 cells have increased CDK2 activity and increased levels of pNPM at the restriction point in G1. As CDK2 activation of NPM1 in S phase promotes centrosome duplication, this suggests that acceleration of the timing of cyclin/CDK cycle activity disrupts the timing of the downstream centrosome duplication cycle. The combined acceleration of the cyclin/CDK/centrosome cycle timing may partially explain the centrosome phenotype observed in *KIF7* depleted RPE cells, as inactivation of *KIF7* was shown to promote premature centrosome duplication when arrested in G1. This also suggests that KIF7s role in regulating these biological processes may be conserved from mice to humans. Further work will be necessary to determine the role of *KIF7* in human cells.

This work provides further evidence for a role for microtubules in regulating cell signaling and the G1/S transition. Previously, a microtubule disruption checkpoint was suggested to exist in the G1 cell cycle phase as treatment with microtubule damaging agents caused cell cycle arrest in G1 and G2/M cell cycle phases. [47,48]. However, the role microtubules play in the G1 phase of the cell cycle is still controversial [49–51]. Earlier work has shown that suppression of microtubule dynamic instability can enhance microtubule-mediated transport and induce the expression and nuclear translocation of the cell cycle protein P53 in human lung A549 cells [50]. Additionally, a recent report has emerged showing that the microtubule-associated protein MARK4 is essential for cytoskeletal maintenance and regulation of the G1/S transition [51]. Based upon these findings, we propose that microtubule dynamics hone cell signaling necessary for proper timing of cell cycle progression, cell cycle exit, and cellular differentiation.

We found that stabilization of microtubules with paclitaxel in G1 synchronized cells leads to misactivation of CDK2, NPM1, inactivation of RB, and increased cyclin d1 expression, all phenotypes similar to what we observed in KIF7 depleted cells.

Depolymerization of microtubules in KIF7 depleted cells within G1 by treatment with nocodazole partially restored the levels of cyclin b, pCDK2, and p21 to levels more similar to control cells, suggesting that these signaling defects occur through disruption of microtubule dynamics. It is possible that microtubules act to directly regulate the stability of these proteins while they are localized in the cytoplasm, in a manner similar to what has been described in mitosis [45]. Alternatively, microtubules may regulate upstream factors that promote the stability or degradation of these molecules. Recent work has shown that proper regulation of microtubule acetylation is necessary to control contact inhibition of cell proliferation, a process intimately related to cell cycle progression [52]. Further work will be needed to determine how cytoskeletal microtubules regulate contact inhibition and cell cycle progression. Our work suggests that KIF7 regulates cytoskeletal microtubule processing in the G1 cell cycle phase, and disruption of normal microtubule function alters cell cycle exit, cell cycle re-entry, and the saturation density of cells in organs such as the lung.

Using confocal microcopy, we determined that KIF7 localizes to the tips of the primary cilia of both undifferentiated epithelial and mesenchymal cells during the branching morphogenesis stage of lung development (Fig 1). Our results suggest that loss of KIF7 disrupts *Hh* signaling within the primary cilium of mesenchymal cells; however, the function of KIF7 and the primary cilium in E14.5 epithelial cells remains unknown. Further work will be necessary to determine if primary cilia help coordinate cell signaling events within epithelial progenitor cells, and if loss of ciliary signaling affects respiratory epithelial cell differentiation.

The respiratory airway is a complex structure containing numerous cell types that possess the ability to communicate with one another and regenerate following injury. Despite the interest in identifying genes and cellular pathways that promote lung regeneration, the molecular mechanisms controlling alveolar epithelial cell homeostasis, remains largely unknown. Further work will be required to identify pathways that promote AEC cell proliferation without disrupting cell exit or differentiation.

## Materials and Methods

### Animals

The generation of Kif7^dda^ congenic mice was previously described [14]. FVB/NJ (JAX 001800) mice were obtained from The Jackson Laboratory and used in the generation of mouse lung fibroblasts. Gli1^LacZ^ (Jax 008211 Gli1^tm2Alj^) mice were a kind gift from Margot Mayer-Proschel (University of Rochester School of Medicine and Dentistry). For BrdU incorporation: 100 ug of BrdU (Sigma)/g of mouse was injected into timed pregnant females 1 hour before embryo harvest. The University Committee on Animal Resources (UCAR) at the University of Rochester Medical Center approved all experimental animal procedures. Euthanasia was performed in accordance with AVMA (American Veterinary Medical Association) guidelines and UCAR Policy on Euthanasia for Rodent Embryos, Fetuses and Neonates. Rodents were euthanized with carbon dioxide followed by cervical dislocation.

### Establishment of Mouse Embryonic Fibroblasts (MEFs), Mouse Lung Fibroblasts (MLFs), and Culture of Mouse Lung Epithelial (MLE) Cells and C3H10T1/2 Cells

#### Generation of MEFs

Embryonic day (E)12.5—E14.5 embryos were collected from timed pregnant female mice. Embryos were dissected free from yolk sacs and the heads were collected for genotyping. After livers were removed, the remaining trunks were minced and digested with 1x trypsin-EDTA (Gibco) for 30 minutes. The resulting cell suspension was filtered though a 40 micron cell strainer (Fisherbrand) and plated in 10 cm dishes. All MEFs used in subsequent experiments were between 2–4 passages unless otherwise stated. Mouse embryonic fibroblasts were cultured in DMEM (Gibco), with 10% fetal bovine serum (Sigma) and 1X antibiotic-antimycotic (Gibco).

#### Generation of mouse lung fibroblasts (MLFs)

E18.5 to post-natal day 3 FVB/NJ embryos/pups were collected and lungs were removed and washed in PBS. Lungs were then minced and resuspended in collagenase (Worthington) in PBS and digested for approximately 1 hour. The cell suspension was passed through a 40 micron cell strainer and plated for 2 hours on 10 cm dishes to allow for the adherence of fibroblasts. Dishes were washed in PBS two times and cells were cultured as described above.

#### C3H10T1/2 cells

C3H10T1/2 cells were obtained from ATCC and maintained as suggested. Cells were cultured in DMEM (Gibco), with 10% fetal bovine serum (Hyclone), and 1X antibiotic-antimycotic (Gibco).

#### Mouse lung epithelial cells (MLE15 cells)

Mouse lung epithelial cells (MLE15 cells) were a gift from Thomas Mariani (University of Rochester School of Medicine and Dentistry) and cultured in DMEM (Gibco), with 10% fetal bovine serum (Hyclone), with nonessential amino acids (Gibco) and 1X antibiotic-antimycotic (Gibco).

### Plasmids

Mouse *Kif7* ShRNA expressing plasmids were purchased from Sigma. KIF7-GFP and KIF7-L657*-GFP were generated by cloning full length *Kif7* and amino acids 1–656 into pEGFP-N1 (ClonTech) using standard procedures. Transfection of GFP expressing plasmids was performed using Lipofectamine 2000 Transfection Reagent (Invitrogen) according to the manufacturer instructions.

### Lentiviral Infections

The viral power lentiviral system was used to generate scrambled control and *Kif7* ShRNA knockdown cell lines. HEK293T cells were transfected using the calcium phosphate transfection kit (Invitrogen). The following day, cells were rinsed in PBS and fresh media was added. 48 hours after transfection, lentivirus containing media was collected, centrifuged at 1000 rpm for 10 minutes, and passed through a .45 uM filter (Thermo). MLE15 cells and MLFs were infected with lentivirus in media with 5 ug/ml polybrene (Sigma). 24 hours after infection, cells were washed in PBS, and the tissue culture media was replaced. The following day cells were treated with 5ug/ml puromycin (Gibco) for 3 days. Knockdown efficiency was determined by western blot analysis.

### Cell Proliferation, Senescence, Density, and Synchronization Assays

#### 3T9 senescence assay

Second passage MEFs were counted and 9*10^5^ cells were plated in 60 mm dishes. After 3 days cells were harvested, counted, and replated as described.

#### Cell proliferation assays

Cell proliferation of MEFs and C3H10T1/2 cells was examined by plating 3*10^5^ cells into 12 well dishes. Cell proliferation of MLE15 cells was examined by plating 5*10^5^ cells in 6 well dishes. Cells were fed every other day.

#### Cell density assay

MLFs were counted and 4.5*10^5^ cells were plated in 12 well plates (day-1). On day 0, several wells were collected and counted to confirm equal plating and the remaining wells were washed and media was replaced with DMEM with 0.5% FBS to induce *Hedgehog* signaling. Cells were collected and counted 3–4 days later.

#### Cell synchronization

For synchronization of cells at the restriction point in G1, cells were washed in PBS and then media was replaced with DMEM with 0.25–0.5% FBS. After 2–3 days cells were washed in PBS, and the media containing 10% serum was added to cells. Synchronization of cells at the G2/M checkpoint was performed by treating cells with 100 ng/mL nocodazole (Sigma) in complete media overnight. The following day, cells were washed in PBS and complete media was added. Cell synchronization was assessed by flow cytometry. Nocodazole and paclitaxel (Sigma) treatment of cells in G1 was performed after synchronizing cells by serum starvation for 2–3 days, and then 10–25 uM paclitaxel (Sigma), or paclitaxel and 30 ug/mL cycloheximide (Sigma) were added for 6–8 hours. Thirty uM Tame-HCl (Tocris) was added to MLE15 cells simultaneously with nocodazole to inhibit Anaphase Promoting Complex (APC) activity.

### Quantification of Microtubule Cytoskeleton Organization and Microtubule Stability Assay

#### Measuring microtubule cytoskeletal shape

Twenty thousand C3H10T1/2 cells were plated on Poly-L-lysine coated glass coverslips in 12 well dishes, and incubated overnight. Cells were immunostained for tubulin and then imaged on a Leica DM5500 B microscope. A length/width measurement of the microtubule cytoskeleton was then measured using ImageJ as described in [32]. The longer side of a cell was measured as length.

#### Microtubule stability assay

Twenty thousand C3H10T1/2 cells were plated and cultured as described above. The assay was carried out as described in the text and in S4 Fig.

### Cell Sorting and Flow Cytometry

Fluorescence activated cell sorting (FACS) cell cycle analysis was performed using the BD Brdu FitC kit according to manufacturer instructions (BD). Cells were collected on a LSR II flow cytometer and the analysis was performed using FlowJo software. DNA content analysis was performed by staining cells with propidium iodide (PI) (Sigma) and analysis by FACS. Briefly, cells were trypsinized, washed in PBS, and fixed overnight at 4 degrees Celsius in 70% ethanol. Cells were washed in PBS and incubated at room temperature with 1 mg/ml RNAase (Sigma) in PBS for 1 hour before treatment with treatment 20 ug/ml PI (Sigma). At least 10,000 events were collected for analysis. Cells were analyzed using FlowJo data analysis software.

### Western Blot Analysis, Cell Fractionation, and Co-IP

Cells were washed in PBS and lysed in 1X RIPA (Millipore) with protease and phosphate inhibitors (Roche). Lysates were cleared by centrifugation, and protein concentration was determined using the Pierce BCA Protein Assay Kit (Thermo Scientific). Fifteen to twenty micrograms of protein was boiled in 2X sample buffer and then resolved on 4–12% gels (Biorad). Protein was transferred to nitrocellulose (GE Healthcare) and blocked in TBS-T (0.1% tween) with 5% milk for at least 1 hour. Primary antibodies were added overnight in blocking buffer. The following days blots were washed for 1 hour in TBS-T, and then secondary antibodies were added in blocking buffer for 1 hour. Blotted protein was visualized using Thermo ECL reagents. Western blots performed on serum starved MEFs were performed by pooling several 10 Cm dishes of approximately 0.7*10^6^ cells (40–60% confluent) control and Kif7 mutant MEFs due to low protein concentrations. Cell fractionation was performed as described by [53]. Co-immunoprecipitation of GFP-fused KIF7 was performed using the GFP trap kit (Chromotek) according to the manufacturer instructions. Quantification of bands from immunoblots was performed using Image J software.

### Histology, Microscopy, and Immunofluorescent Staining

#### Histology

Hematoxylin and Eosin, Elastin staining, and Beta-galactosidase staining of tissue sections were performed using standard procedures. For the collecting of timed pregnant embryos, day 0.5 was considered as noon of the day of detection of a copulation plug. Tissues were fixed in 4% PFA overnight, embedded in paraffin wax, and sectioned at 5 uM for histology and immunofluorescent staining. Beta-galactosidase staining was performed on 10 uM frozen sections.

#### Microscopy

Transmission electron microscopy was performed using a Hitachi 7650 microscope on whole lungs dissected from E18.5 mutant embryos at the University of Rochester Medical Center (URMC) Electron Microcopy core. Lungs were fixed in buffered 2.5% glutaraldehyde, post-fixed in 1% osmium tetroxide, dehydrated, infiltrated and embedded into EPON/Araldite resin.

An Olympus FV1000 laser scanning confocal microscope was used to capture confocal images at the URMC Confocal microscopy core. A Leica MZ10F stereoscope, a Leica DM5500 B microscope, and Leica DFC 365FX and Leica MC170 HD cameras were used for conventional microscopy. Images were converted to jpegs, and contrast and brightness (of entire images) were adjusted using Image J software.

#### Immunofluorescent staining of tissue sections

Tissue sections were dewaxed, rehydrated, and heat mediated antigen retrieval was performed when necessary. Tissue sections were permeabilized in TBS-T (0.5% Triton), blocked in TBS-T (0.01% Tween) with 5% milk, and then stained overnight with primary antibodies. Sections were then washed in TBS-T (0.01% Tween) and then incubated with secondary antibodies for 1 hour. Hoechst 33342 (Thermo) was used to stain nuclei.

#### Immunofluorescent staining of cultured cells

Cover slips with adherent cells were washed in PBS and then fixed in 100% methanol at -20 degrees Celsius for at least 15 minutes. Cells were blocked in TBS-T (0.01% Tween) with 5% milk and then stained overnight with primary antibodies. The following day, cells were washed in TBS-T and incubated with secondary antibodies.

#### Quantification of immunopositive cells *in vivo*

Tissue sections from control and *Kif7* mutant lungs were immunostained and photographed as described above. At least 4 consecutive tissue sections from similar planes of control and mutant lungs were counted and averaged to generate a single biological sample. At least 4 sets of control and mutant lungs from at least 3 independent litters were used for our analysis.

### Antibodies

The following antibodies and dilutions were used in this study: Rb anti KIF7 1:1000 (a gift from Kathryn Anderson, Rb anti Gli1 1:5000 (Novus Biologicals), Rb anti Cyclin D1 1:200 (Pierce), Rb anti Cyclin E1 1:500 (Abcam), Rb anti actin 1:5000 (Santa Cruz), Ms anti B tubulin 1:1000 (developmental hybridoma bank), Ms anti E Candherin 1:300 (BD), Ms anti Acetylated alpha tubulin 1:1000 (Sigma), Hm anti Podplanin 1:500 (Abcam), Ms anti Fasn 1:300 (Santa Cruz), Rb anti Ki67 1:300 (Abcam), Rb anti Gli2 1:1000 (Abcam), Rb anti Gli3 1:500 (Santa Cuz), Rb and pNpm^Thr199^ 1:1000 (Cell Signaling), Rb anti Detyrosinated alpha tubulin 1:5000 (Millipore), Ms anti A tubulin 1:1000 (Sigma), Rb anti Phos-histone H3 1:300 (Millipore), Rb anti HH3 1:1000 (Cell Signaling), Rat anti Brdu 1:500 (Abcam), Rb anti SftpC 1:200 (Santa Cruz), Rb anti p27 1:1000 (Santa Cruz), Rb anti pRb^S807/811^ 1:2500 (Cell Signaling), Rb anti pCdk2^T160^ 1:1000 (Cell Signaling), Rb anti Cyclin A 1:1000 (Santa Cruz), Rb anti Cyclin B 1:1000 (Cell Signaling), Rb anti p21 1:500 (Santa Cruz), Gt anti Y tubulin 1:200 (Santa Cruz), Rb anti pCdk1/Cdc2^T161^ 1:1000 (Cell signaling), Ms anti GFP 1:500 (Santa Cruz), Rb anti Fzr 1:2000 (Invitrogen), Rb anti p55^cdc^ 1:1000 (Santa Cruz), Rb anti pErk1/2^T202^ 1:2000 (Cell Signaling), Rb anti Erk1/2 1:3000 (Cell Signaling), and Rb anti pAkt^S473^ 1:2000 (Cell Signaling). Secondary HRP conjugated antibodies were used from Santa Cruz, and Alexa Fluor fluorescent secondary antibodies were purchased from Invitrogen.

### Real Time Quantitative Polymerase Chain Reaction (RT-qPCR)

RNA was isolated from tissue or cells using the Qiagen RNA easy kit. E18.5 control and *Kif7* mutant lungs were perfused with 1 ml of cold PBS and then mechanically disrupted in RLT lysis buffer. RNA isolation was carried out according to the manufacturer instructions. CDNA was reverse transcribed using 0.5–1.0 ug of RNA using the BioRad cDNA synthesis kit. RT-qPCR was performed on BioRad icycler using gene specific RT-qPCR primers obtained from the Mass General Primer Bank. All RT-qPCR reactions were performed at temperatures determined to generate a 90–100% reaction efficiency. All reactions were performed with at least 2 technical replicates and 3–6 biological replicates.

### Statistical Analysis

All experiments were performed with at least 3 biological replicates unless otherwise stated. P values for statistical analysis were calculated using a two-tailed Students T test. P values of less than 0.05 were considered significant. All graphs were generated in Excel, and error bars represent SEM.

## Supporting Information

S1 Fig KIF7 expression is lost in *Kif7* ^*dd/dd*^ mutant lungs. (**A.-F.**) Confocal co-immunofluorescent staining of KIF7 with acetylated alpha tubulin in the respiratory epithelium of E14.5 control and *Kif7* ^*dd/dd*^ mutant lungs. (**A.-B.**) The scale bar is 50 microns in **A-B**, and 10 microns in **C-D**. **E+F** are zooms of the boxed regions in **C+D**.
(TIF)

S2 Fig *Kif7* mutant lungs are dense and hypercellular. (**A.+A’**) Hematoxylin and eosin (H+E) stained tissue sections of E18.5 control (**A**) and *Kif7* ^*dd/dd*^ mutant lungs (**A’**). (**B.+B’**) Elastin staining (black) of E18.5 control and *Kif7* ^*dd/dd*^ mutant lungs. Scale bar is 25 microns. (**C.**) Real-time quantitative polymerase chain reaction (RT-qPCR) analysis of transcript levels in E18.5 *Kif7* ^*dd/dd*^ mutant and littermate control lungs. N≥3* P<0.05. (**D.**) Quantification of number of nuclei/20x field from H+E stained tissue sections. N≥3, * P<0.05, **P<0.01.
(TIF)

S3 Fig *Kif7* mutant MEFs have an increased growth rate and increased growth potential. (**A.-B.**) Growth curve and senescence assay of control and *Kif7* ^*dd/dd*^ mutant mouse embryonic fibroblasts (MEFs). Growth curves were performed on cells pooled from genotyped embryos. All curves are representative of multiple independent experiments. (**C.-C’**) Crystal violet stained nuclei of serum starved post-confluent control (**C.**) and *Kif7* ^*dd/dd*^ mutant (**C’**) MEFs. Scale bar is 1.5 mm. (**D.**) MEFs were arrested at 50% confluency by serum depravation and western blot analysis was performed on protein lysates. (**E.**) Cell cycle analysis was performed by propidium staining and flow cytometry on preconfluent serum starved MEFs to confirm that cells were arrested at the restriction point in G1. (**F.-F’**) Immunofluorescent staining for B tubulin and cyclin d1 in G1 synchronized control (**F.**) and *Kif7* ^*dd/dd*^ mutant (**F’**) MEFs. Scale bar is 40 microns. (**G.**) Quantification of the percent of cyclin d1 positive MEFs. 50–100 cells were counted and then averaged from at least 3 independent fields in at least 3 independent experiments. N≥3 * P<0.05.
(TIF)

S4 Fig Strategy for measuring microtubule stability in C3H10T1/2 cells. (**A.**) Cells were washed in PBS, and then incubated with ice cold PBS for 45 minutes, while plates were submerged in ice. The PBS was removed and replaced with ice cold methanol. Plates were maintained at room temperature for approximately 15 minutes before placing at -20°C for approximately 30 minutes. Plates for time 0 were collected and fixed with methanol before processing for ICC to visualize microtubule polymers. (**B.-E.**) Representative photographs of B tubulin staining at time 0 and after 45 minutes on ice. (**F.+G.**) Co-immunofluorescent staining of B tubulin and F-actin (phalloidin) staining in control and KIF7 depleted C3H10T1/2 cells. Cells were fixed in 4%PFA to preserve actin, therefore microtubule staining may appear differently than in **B.-E.** (**H.**) Quantification of cell area based up F-actin staining. Image J was used to measure the area of at least 25 cells from 4 independent experiments.
(TIF)

S5 Fig Characterization of type II AECs *in vivo* and MLE15 cells *in vitro*. (**A’-G.**) Confocal co-immunofluorescent staining. (**A’-A”‘)** Co-immunofluorescent staining of tissue sections from E18.5 lungs for surfactant protein c (SFTPC) and acetylated alpha tubulin. (**B.**) Co- immunofluorescent staining of tissue sections from E13.5 lungs for acetylated alpha tubulin. Note that E18.5 SFTPC+ cells do form primary cilia, while undifferentiated E13.5 lung epithelial cells form primary cilia. (**C.-E.**) MLE15 cells were synchronized in G1 by serum starvation and immnostained for CDH1 (e cadherin), alpha tubulin, and acetylated alpha tubulin. (**F.**) Acetylated alpha tubulin staining in primary cilia of G1 synchronized mouse lung fibroblasts. Note that MLE15 cells fail to form primary cilia (**E.**), unlike mouse lung fibroblasts (**F.**). (**G.**) Co-immunofluorescent staining for acetylated alpha tubulin and gamma tubulin (a centrosome marker) in MLE15 cells. Note that acetylated alpha tubulin is expressed within the centrosome, and not in ciliary like structures of MLE15 cells. The scale bar is 10 microns in **A.-E.** and 1 micron in zooms of the centrosome in **E.**. (**H.-I.**) Real-time quantitative polymerase chain reaction analysis of transcript levels in serum starved MLE15 cells and mouse lung fibroblasts before and after the addition of the Smo/Hh agonist SAG. Note that only the mouse lung fibroblasts are competent to induce the expression of *Hh* target gene *Gli1*. (**J.**)Western blot analysis of serum starved Scram control and KIF7 depleted MLE15 cells. Note that depletion of KIF7 protein does not affect GLI1 or GLI3R levels in MLE cells. We were unable to detect GLI2 protein under these conditions in MLE15 cells. N≥3, * P<0.05, **P<0.01.
(TIF)

S6 Fig Flow cytometric analysis of G1 synchronized MLE15 cells. (**A.**) Western blots of protein lysates from asynchronous control or KIF7 depleted MLE15 cells. (**B.**) Contour map of the cell cycle profile of control and *Kif7* depleted MLE15 cells following G1 synchronization (by serum starvation) and after the readtion of serum containing media. Cells were incubated with BrdU in media either with or without serum and the processed for analysis of BrdU incorporation and total DNA content. Analysis was performed using FlowJo software on five biological replicates.
(TIF)

S7 Fig Co-immunoprecipitation of KIF7-GFP from MLE15 cells. (**A.**) Co-immunoprecipitation experiments with KIF7-GFP expressing asynchronous MLE15 cells. Immunoblots were performed following immunoprecipitation of KIF7-GFP using GFP-trap conjugated to agarose beads. Agarose beads were used as a negative control. *, is a non-specific band. A specific interaction could not be detected between KIF7-GFP and these cell cycle proteins.
(TIF)
