# Supplementary figures and images for: KIF7 Controls the Proliferation of Cells of the Respiratory Airway through Distinct Microtubule Dependent Mechanisms
Source: PLoS Genet. 2015 Oct 6;11(10):e1005525. doi: 10.1371/journal.pgen.1005525 (PMC4595342; doi:10.1371/journal.pgen.1005525)

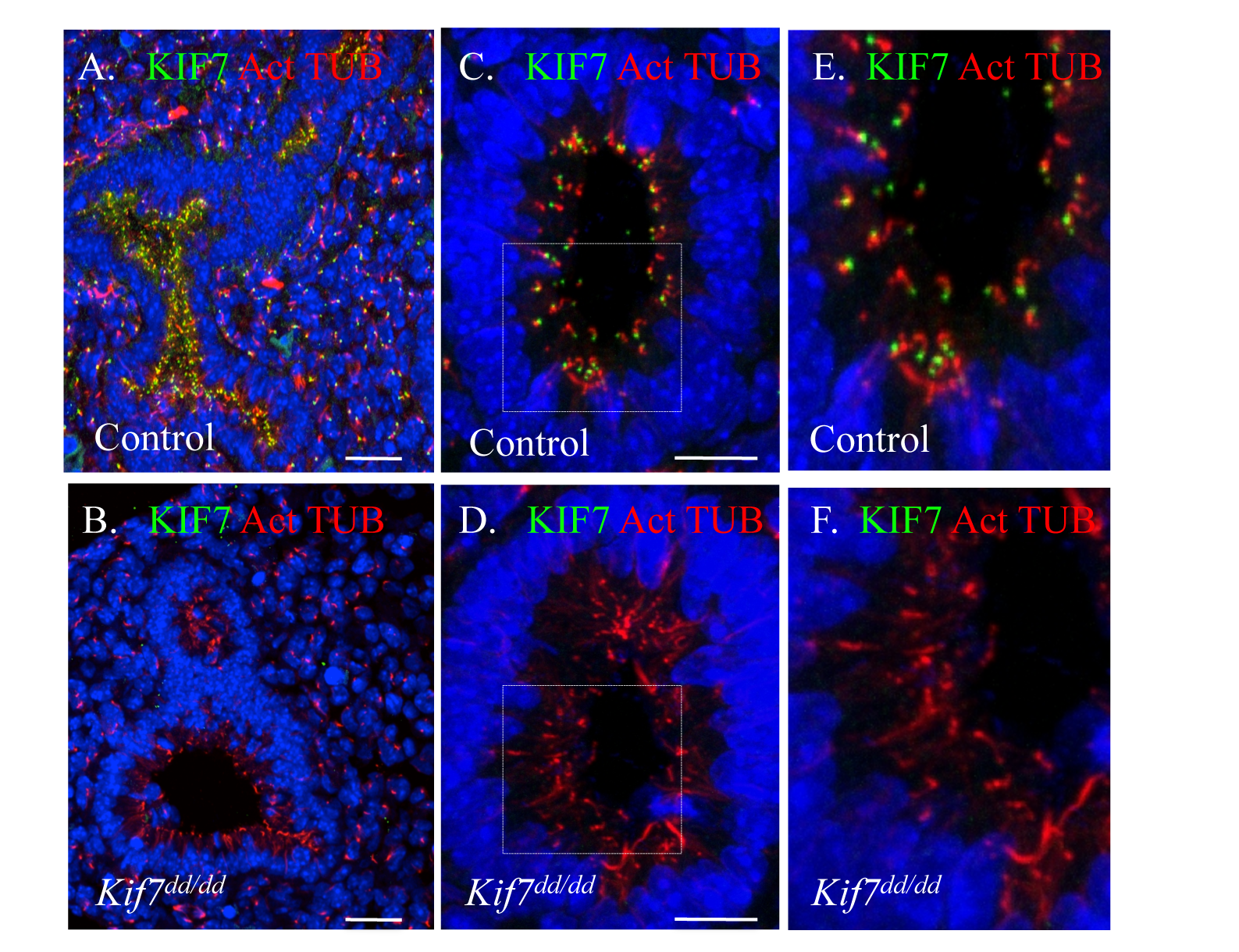

Supplement: S1 Fig — (A.-F.) Confocal co-immunofluorescent staining of KIF7 with acetylated alpha tubulin in the respiratory epithelium of E14.5 control and Kif7 dd/dd mutant lungs. (A.-B.) The scale bar is 50 microns in A-B, and 10 microns in C-D. E+F are zooms of the boxed regions in C+D. (TIF) [file pgen.1005525.s001.tif]

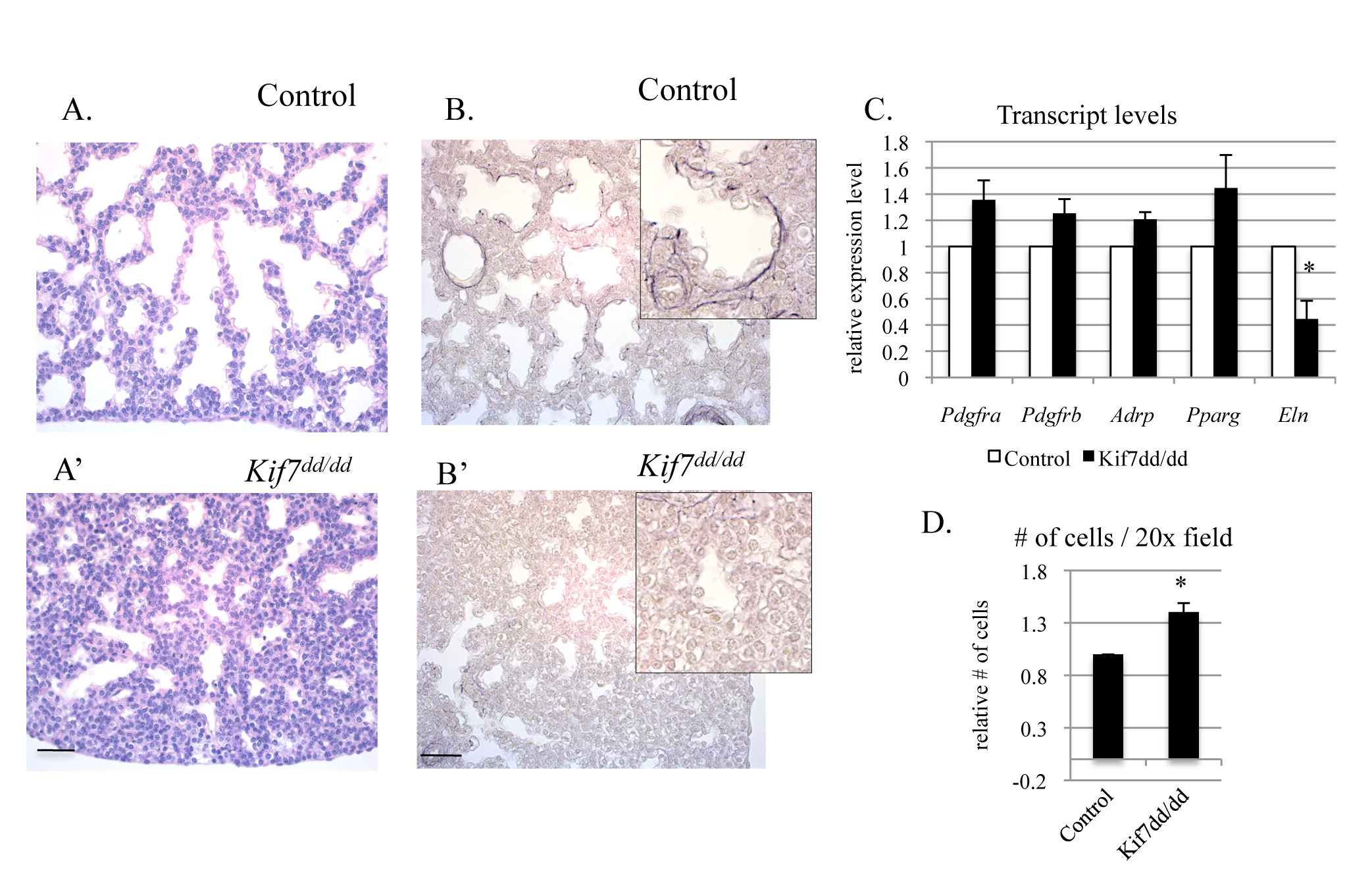

Supplement: S2 Fig — (A.+A’) Hematoxylin and eosin (H+E) stained tissue sections of E18.5 control (A) and Kif7 dd/dd mutant lungs (A’). (B.+B’) Elastin staining (black) of E18.5 control and Kif7 dd/dd mutant lungs. Scale bar is 25 microns. (C.) Real-time quantitative polymerase chain reaction (RT-qPCR) analysis of transcript levels in E18.5 Kif7 dd/dd mutant and littermate control lungs. N≥3* P<0.05. (D.) Quantification of number of nuclei/20x field from H+E stained tissue sections. N≥3, * P<0.05, **P<0.01. (TIF) [file pgen.1005525.s002.tif]

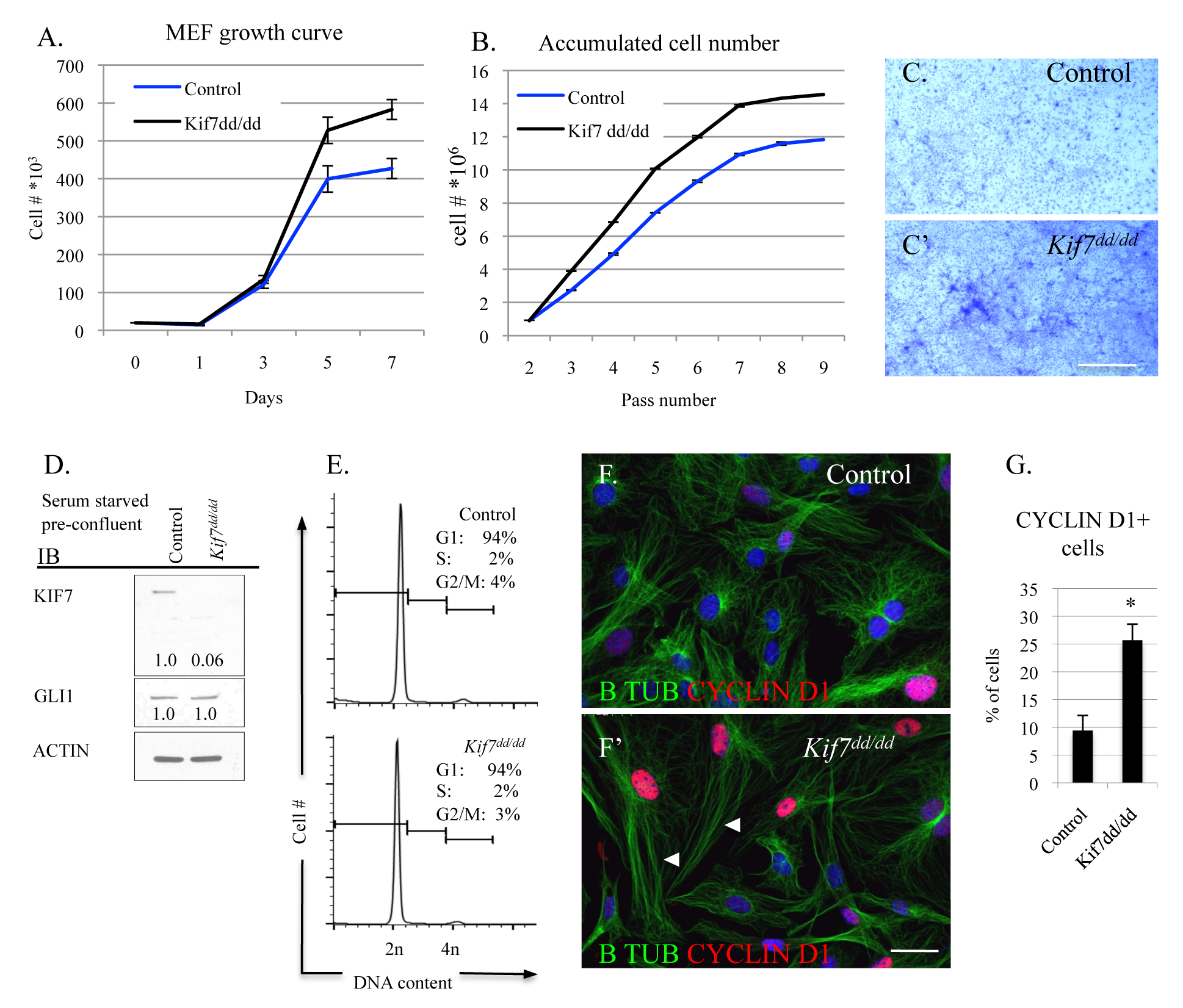

Supplement: S3 Fig — (A.-B.) Growth curve and senescence assay of control and Kif7 dd/dd mutant mouse embryonic fibroblasts (MEFs). Growth curves were performed on cells pooled from genotyped embryos. All curves are representative of multiple independent experiments. (C.-C’) Crystal violet stained nuclei of serum starved post-confluent control (C.) and Kif7 dd/dd mutant (C’) MEFs. Scale bar is 1.5 mm. (D.) MEFs were arrested at 50% confluency by serum depravation and western blot analysis was performed on protein lysates. (E.) Cell cycle analysis was performed by propidium staining and flow cytometry on preconfluent serum starved MEFs to confirm that cells were arrested at the restriction point in G1. (F.-F’) Immunofluorescent staining for B tubulin and cyclin d1 in G1 synchronized control (F.) and Kif7 dd/dd mutant (F’) MEFs. Scale bar is 40 microns. (G.) Quantification of the percent of cyclin d1 positive MEFs. 50–100 cells were counted and then averaged from at least 3 independent fields in at least 3 independent experiments. N≥3 * P<0.05. (TIF) [file pgen.1005525.s003.tif]

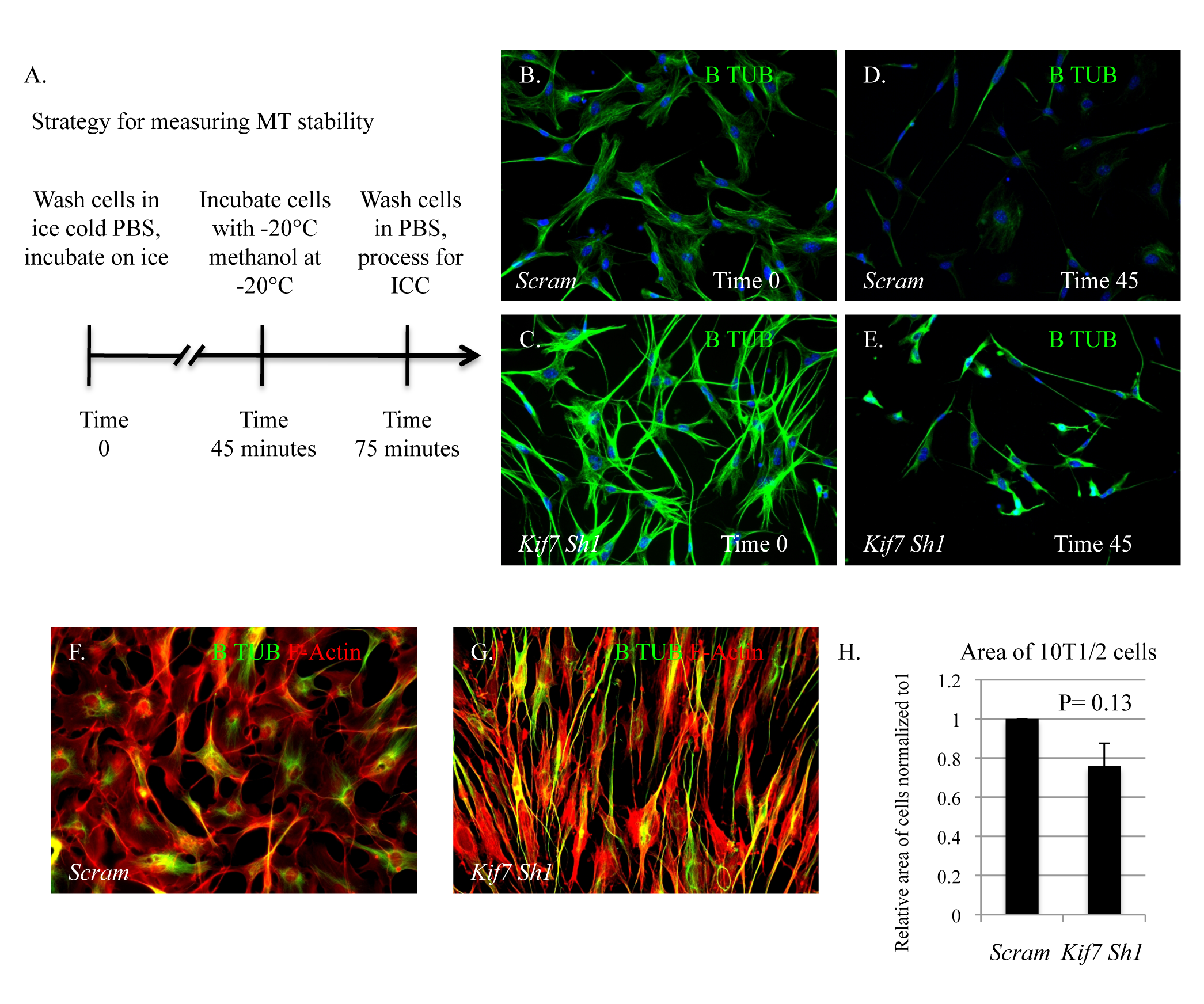

Supplement: S4 Fig — (A.) Cells were washed in PBS, and then incubated with ice cold PBS for 45 minutes, while plates were submerged in ice. The PBS was removed and replaced with ice cold methanol. Plates were maintained at room temperature for approximately 15 minutes before placing at -20°C for approximately 30 minutes. Plates for time 0 were collected and fixed with methanol before processing for ICC to visualize microtubule polymers. (B.-E.) Representative photographs of B tubulin staining at time 0 and after 45 minutes on ice. (F.+G.) Co-immunofluorescent staining of B tubulin and F-actin (phalloidin) staining in control and KIF7 depleted C3H10T1/2 cells. Cells were fixed in 4%PFA to preserve actin, therefore microtubule staining may appear differently than in B.-E. (H.) Quantification of cell area based up F-actin staining. Image J was used to measure the area of at least 25 cells from 4 independent experiments. (TIF) [file pgen.1005525.s004.tif]

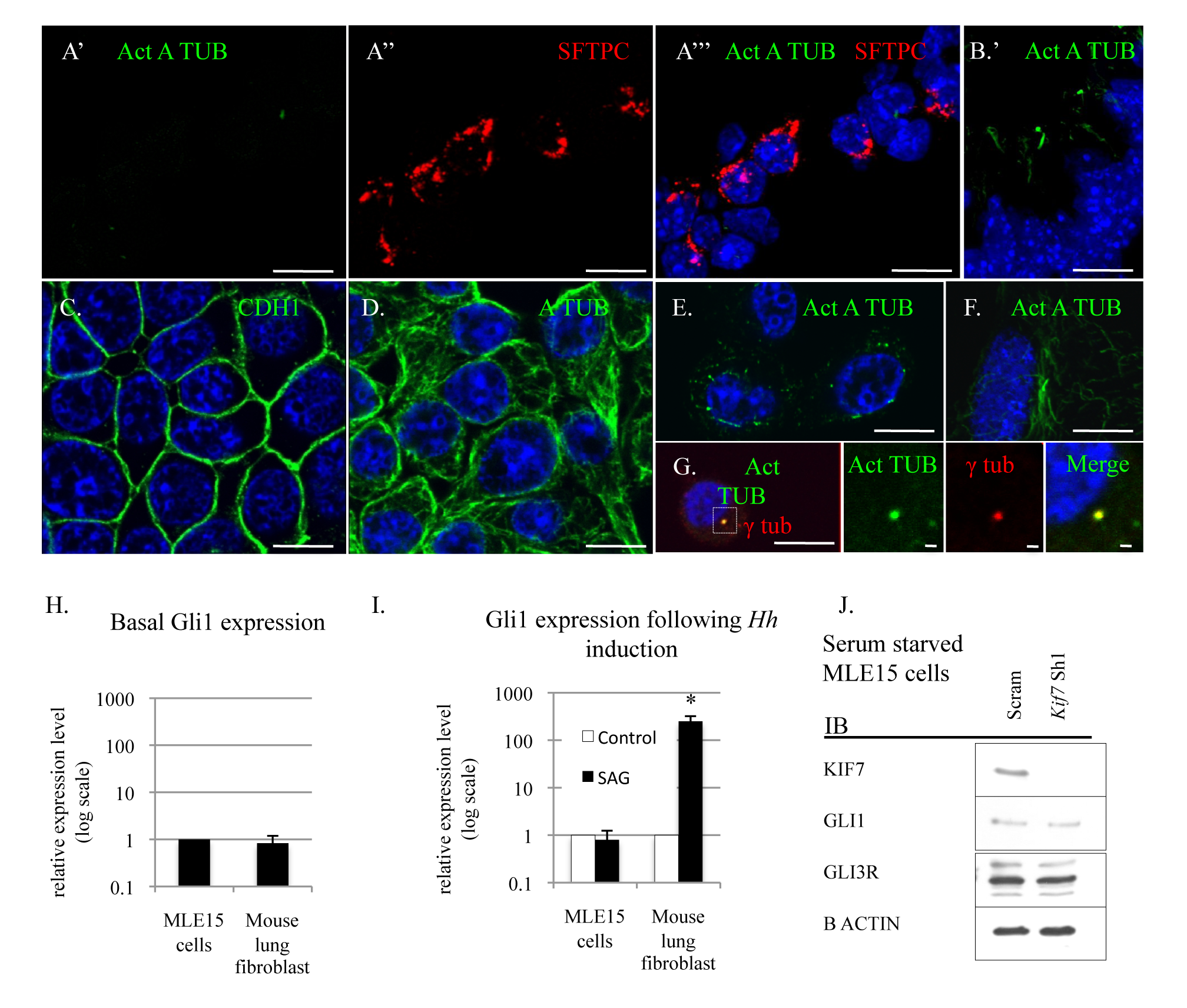

Supplement: S5 Fig — (A’-G.) Confocal co-immunofluorescent staining. (A’-A”‘) Co-immunofluorescent staining of tissue sections from E18.5 lungs for surfactant protein c (SFTPC) and acetylated alpha tubulin. (B.) Co- immunofluorescent staining of tissue sections from E13.5 lungs for acetylated alpha tubulin. Note that E18.5 SFTPC+ cells do form primary cilia, while undifferentiated E13.5 lung epithelial cells form primary cilia. (C.-E.) MLE15 cells were synchronized in G1 by serum starvation and immnostained for CDH1 (e cadherin), alpha tubulin, and acetylated alpha tubulin. (F.) Acetylated alpha tubulin staining in primary cilia of G1 synchronized mouse lung fibroblasts. Note that MLE15 cells fail to form primary cilia (E.), unlike mouse lung fibroblasts (F.). (G.) Co-immunofluorescent staining for acetylated alpha tubulin and gamma tubulin (a centrosome marker) in MLE15 cells. Note that acetylated alpha tubulin is expressed within the centrosome, and not in ciliary like structures of MLE15 cells. The scale bar is 10 microns in A.-E. and 1 micron in zooms of the centrosome in E.. (H.-I.) Real-time quantitative polymerase chain reaction analysis of transcript levels in serum starved MLE15 cells and mouse lung fibroblasts before and after the addition of the Smo/Hh agonist SAG. Note that only the mouse lung fibroblasts are competent to induce the expression of Hh target gene Gli1. (J.)Western blot analysis of serum starved Scram control and KIF7 depleted MLE15 cells. Note that depletion of KIF7 protein does not affect GLI1 or GLI3R levels in MLE cells. We were unable to detect GLI2 protein under these conditions in MLE15 cells. N≥3, * P<0.05, **P<0.01. (TIF) [file pgen.1005525.s005.tif]

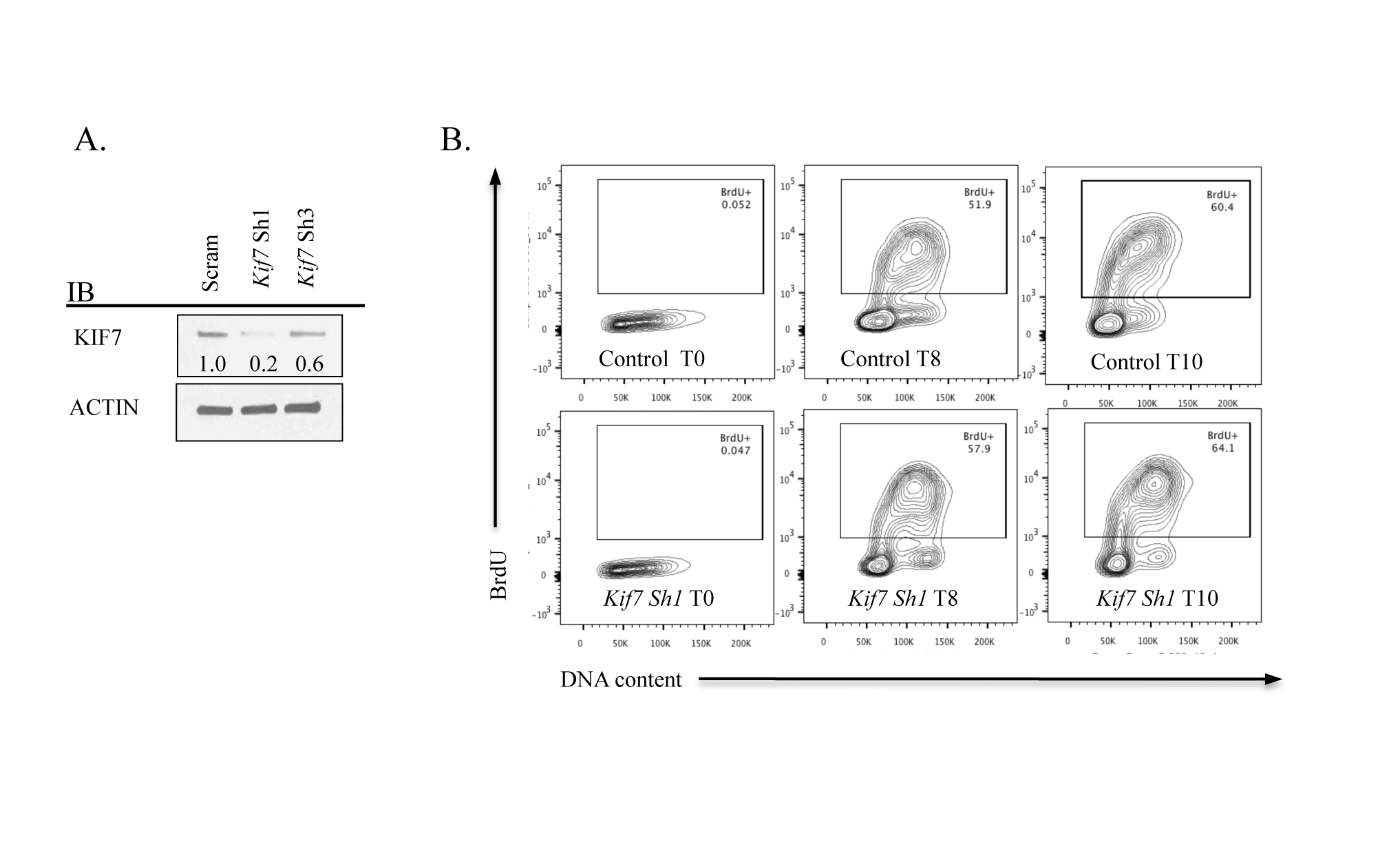

Supplement: S6 Fig — (A.) Western blots of protein lysates from asynchronous control or KIF7 depleted MLE15 cells. (B.) Contour map of the cell cycle profile of control and Kif7 depleted MLE15 cells following G1 synchronization (by serum starvation) and after the readtion of serum containing media. Cells were incubated with BrdU in media either with or without serum and the processed for analysis of BrdU incorporation and total DNA content. Analysis was performed using FlowJo software on five biological replicates. (TIF) [file pgen.1005525.s006.tif]

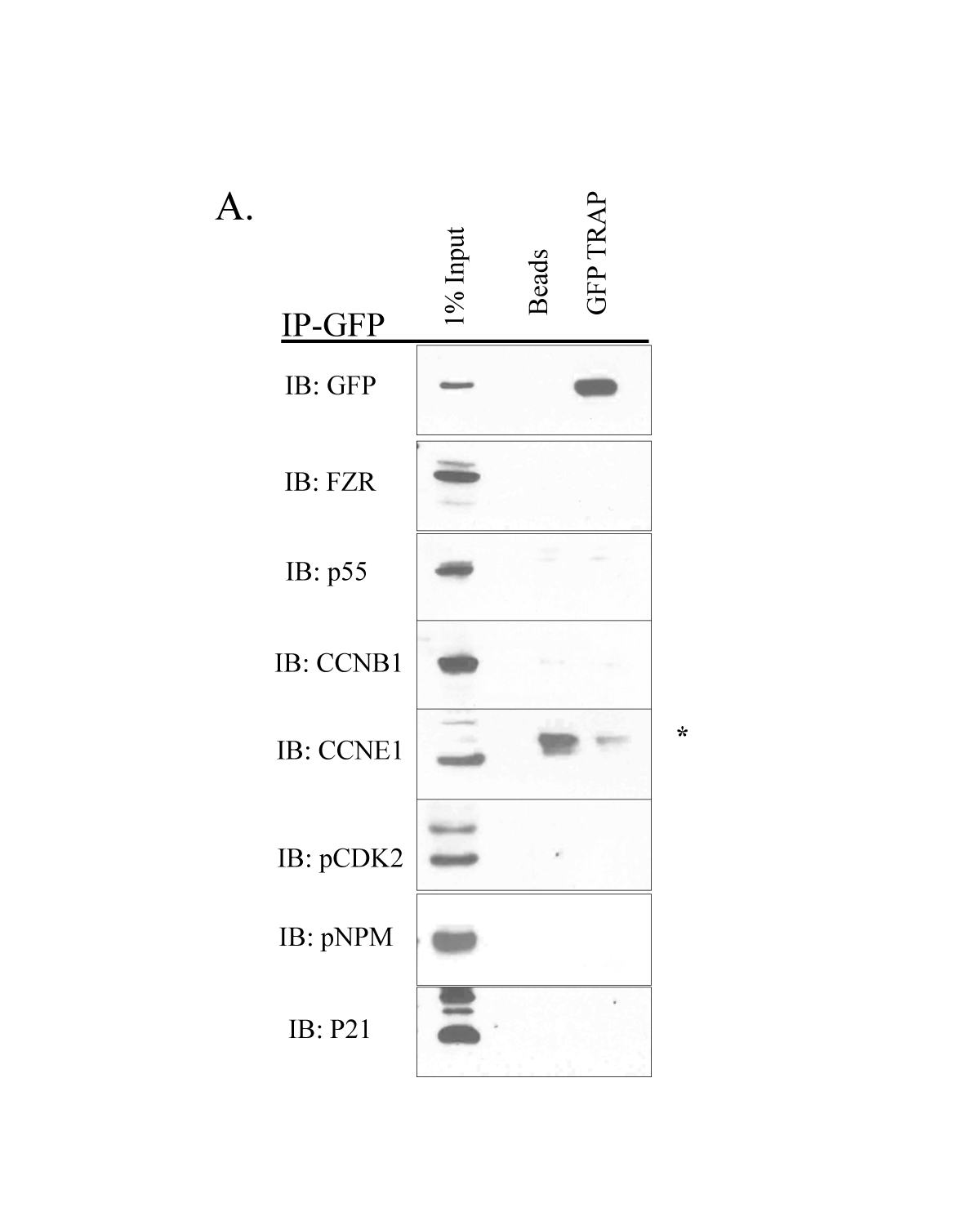

Supplement: S7 Fig — (A.) Co-immunoprecipitation experiments with KIF7-GFP expressing asynchronous MLE15 cells. Immunoblots were performed following immunoprecipitation of KIF7-GFP using GFP-trap conjugated to agarose beads. Agarose beads were used as a negative control. *, is a non-specific band. A specific interaction could not be detected between KIF7-GFP and these cell cycle proteins. (TIF) [file pgen.1005525.s007.tif]
